# Iron Homeostasis and Liver Diseases

**DOI:** 10.1111/jcmm.71412

**Published:** 2026-10-21

**Authors:** Peng Yang, Zhitian Shi, Qin Huang, Zhihong Tu, Yue Sun, Lin Wang, Yonglong Wei

**Affiliations:** ^1^ School of Life Sciences, Yunnan Key Laboratory of Cell Metabolism and Diseases Yunnan University Kunming Yunnan China; ^2^ Department of Hepatopancreatobiliary Surgery The Second Affiliated Hospital of Kunming Medical University Kunming Yunnan China; ^3^ Southwest United Graduate School Kunming Yunnan China; ^4^ Key Laboratory of Tumor Immunological Prevention and Treatment of Yunnan Province Kunming Yunnan China

**Keywords:** ferroptosis, hepatocellular carcinoma, iron homeostasis, liver fibrosis, metabolic dysfunction‐associated steatotic liver disease

## Abstract

Iron is an essential micronutrient whose ability to cycle between ferric (Fe^3+^) and ferrous (Fe^2+^) states makes it crucial for physiological processes, including oxygen transport, DNA synthesis, and oxidative phosphorylation. However, because both deficiency and excess can cause severe pathology, precise regulation is critical. The liver plays a central role in systemic iron metabolism, and growing evidence identifies hepatic iron dyshomeostasis as a key catalyst in diverse liver disorders, including metabolic dysfunction‐associated steatotic liver disease (MASLD/MASH), alcoholic liver disease, liver fibrosis, and hepatocellular carcinoma (HCC). Hepatic iron accumulation promotes liver injury primarily through oxidative stress, mitochondrial dysfunction, and ferroptosis. In this review, we synthesize the multi‐layered regulatory landscape of hepatic iron homeostasis, encompassing systemic coordination, cellular fine‐tuning, and epigenetic modifications. We thoroughly dissect the pathogenic impact of iron overload and critically evaluate emerging therapeutic strategies aimed at restoring iron balance, such as hepcidin mimetics and ferroptosis modulators. Finally, we discuss current literature gaps—including preclinical model limitations, the lack of standardized non‐invasive biomarkers, and drug delivery bottlenecks—providing a clear roadmap for future translational research in iron‐related liver pathologies.

## 1 Introduction

Iron is an essential trace element that plays a central role in critical biological processes, including oxygen transport, oxidative phosphorylation, and cell proliferation [1, 2]. To balance supply and demand, systemic iron homeostasis is tightly regulated. Under physiological conditions, daily intestinal iron absorption is strictly limited to 1–2 mg [3]. As the primary hub for iron storage and mobilization [4], the liver is highly susceptible to such homeostatic disruptions. These imbalances underlie hereditary hemochromatosis (HH), which dramatically increases the risk of hepatocellular carcinoma (HCC) [5, 6, 7]. Furthermore, iron dysregulation serves as a key driver in the pathogenesis of various acquired chronic liver diseases.

In recent years, research on iron overload disorders has expanded significantly. Systemic iron homeostasis is primarily governed by the liver‐derived hormone hepcidin. Hepcidin binds to the cellular exporter SLC40A1 (ferroportin) to induce its degradation, effectively restricting iron absorption and macrophage iron release. Abnormal hepcidin regulation and ectopic iron accumulation are now recognized as critical cofactors that exacerbate metabolic dysfunction‐associated steatotic liver disease (MASLD), alcoholic liver disease (ALD), and their severe progressive form, MASH [8, 9]. Beyond these classical hormonal feedback loops, recent studies reveal a deeper layer of control. Prolonged environmental stressors can profoundly reprogram the expression of iron import, storage, and export genes through epigenetic mechanisms, including DNA methylation and histone modifications [10, 11]. This epigenetic fine‐tuning acts as a sophisticated molecular bridge, allowing cells to dynamically adapt to fluctuations in iron availability.

Intracellular iron serves as the primary catalyst for lethal lipid peroxidation; consequently, the aforementioned dysregulation of iron homeostasis fundamentally dictates cellular susceptibility to ferroptosis. As an iron‐dependent form of regulated cell death, ferroptosis plays a complex, dual role in liver pathology. On one hand, it mediates acute liver injury and drives the fibrotic progression of chronic conditions such as MASLD and ALD. On the other hand, ferroptosis represents a distinct therapeutic vulnerability in HCC [12]. Given these critical impacts, decoding the independent yet interconnected networks governing both iron metabolism and ferroptosis is essential. Targeting these pathways has emerged as a highly promising strategy to simultaneously halt chronic liver disease progression and inhibit tumour proliferation.

Although the clinical link between iron dyshomeostasis and liver disease is well established, specific therapies targeting iron metabolism remain limited by the sheer complexity of these regulatory networks. To bridge this gap, this review revisits the fundamental mechanisms of iron homeostasis—encompassing absorption, distribution, and key transport pathways like SLC40A1—while integrating recent advances to highlight frontier areas in iron regulation. Specifically, we systematically examine four core domains: (1) the interplay of critical signalling pathways (e.g., IRP‐IRE, BMP‐SMAD, and IL‐6‐STAT3) governing iron homeostasis; (2) the emerging role of epigenetic mechanisms in fine‐tuning cellular iron metabolism; (3) the distinct role of ferroptosis in liver pathologies, particularly MASLD, MASH, and HCC; and (4) recent therapeutic strategies targeting iron dyshomeostasis in these contexts. By mapping these intricate molecular networks, this review provides a novel perspective on iron‐related metabolic disorders and establishes a robust theoretical foundation for innovative precision therapies, such as interventions concurrently targeting epigenetic pathways and ferroptosis.

## 2 The Functions of Iron

By readily alternating between oxidation states, iron serves as an indispensable mediator of biological electron transfer. At the cellular level, it provides the fundamental substrate for the biosynthesis of heme and mitochondrial iron–sulphur (Fe–S) clusters. These core components are critical for maintaining the respiratory chain, redox homeostasis, and DNA replication. Furthermore, iron crucially modulates immune cell differentiation and polarization (Table 1).

**TABLE 1:** Physiological roles of iron.

Iron function	Description	References
Heme and ISC synthesis	Iron serves as the central metal in heme and forms functional [2Fe–2S] and [4Fe–4S] clusters with sulphur.	[13, 14, 15, 16]
Genomic stability	The nuclease/helicase DNA2, B‐family DNA polymerases—including Pol α, Pol δ, Pol ε, and Pol ζ.	[17, 18, 19, 20, 21, 22]
Cellular respiration and redox homeostasis	Heme‐containing proteins, including electron transport chain cytochromes and catalase, facilitate mitochondrial energy generation and oxidative balance.	[23, 24, 25]
Immune effector molecules	NOS and NOX contain heme and generate immune effector molecules including NO and superoxide anions.	[26, 27, 28, 29, 30]
Adaptive immunity	Excess iron can suppress the proliferation of B and T lymphocytes and inhibit the differentiation of CD4^+^ T cells toward T_H_1, whereas iron deficiency promotes differentiation toward T_H_2.	[31, 32, 33]
Macrophage polarization regulation	High iron levels promote pro‐inflammatory M1 macrophages, whereas low iron levels favour anti‐inflammatory M2 macrophages.	[34, 35, 36, 37, 38, 39]

Abbreviations: ISC, iron–sulphur clusters; NO, nitric oxide; NOS, nitric oxide synthase; NOX, NADPH oxidase.

### 2.1 Heme Iron in Oxygen Binding and Transport

Iron serves as the central element of heme in haemoglobin and myoglobin, proteins essential for sustaining respiratory function [40]. Heme biosynthesis is a highly conserved enzymatic pathway that begins with the condensation of glycine and succinyl‐CoA. Following coordinated reactions across the cytosol and mitochondria, the final step occurs intramitochondrially, where ferrochelatase inserts Fe^2+^ into protoporphyrin IX to yield mature heme [41, 42]. Upon O_2_ binding, partial electron transfer from Fe^2+^ to O_2_ draws the iron into the porphyrin plane. This induces conformational changes within the haemoglobin subunit to drive cooperative oxygen binding. Conversely, in hypoxic tissues, oxygen dissociation triggers a reverse conformational shift, lowering the affinity of remaining subunits to facilitate efficient O_2_ release [43].

Unlike haemoglobin, monomeric myoglobin contains a single heme group and thus lacks cooperative oxygen binding [40, 43]. Myoglobin exhibits a significantly higher oxygen affinity (P_50_ = 2.8 mmHg) [44] than haemoglobin (P_50_ = 26 mmHg) [45]. Consequently, its oxygen dissociation curve is hyperbolic rather than sigmoidal, reflecting myoglobin's primary role in cellular oxygen storage rather than systemic transport [46].

### 2.2 Heme and Fe–S Clusters in Mitochondrial Bioenergetics

Cellular iron utilization is primarily reflected in heme synthesis and the assembly of mitochondrial and cytosolic iron–sulphur clusters. In the cytosol, iron exists mainly as ferrous iron (Fe^2+^) within the labile iron pool (LIP). It is imported into mitochondria predominantly via the mitoferrin transporters SLC25A37 (MFRN1) and SLC25A28 (MFRN2) [47]. Recent studies suggest that mitochondria may also acquire iron through a ‘kiss‐and‐run’ mechanism, delivering iron locally via transient membrane tethering rather than through diffusion or dedicated channels [48, 49]. Once inside the mitochondrial matrix, Fe^2+^ acts as a cofactor for two major biosynthetic pathways. During heme biosynthesis, ferrochelatase catalyzes the insertion of Fe^2+^ into protoporphyrin IX. Concurrently, in Fe–S cluster biogenesis, Fe^2+^ combines with cysteine‐derived sulphur (via NFS1‐mediated desulphurization) and assembles onto the ISCU scaffold protein to form [2Fe–2S] or [4Fe–4S] clusters. Critical chaperones, including GLRX5, ISCA1, and ISCA2, then coordinate cluster maturation and stabilization. Intermediates generated by the mitochondrial ISC system—likely sulphur‐containing compounds rather than intact clusters—are exported across the inner mitochondrial membrane via the ABCB7 transporter [13, 14, 15, 16]. These exported species provide substrates for the cytosolic iron–sulphur assembly (CIA) machinery. This system subsequently facilitates the incorporation of Fe–S clusters into nuclear and cytosolic proteins, including enzymes critical for DNA metabolism and redox regulation [50, 51, 52] (Figure 1).

**FIGURE 1:**
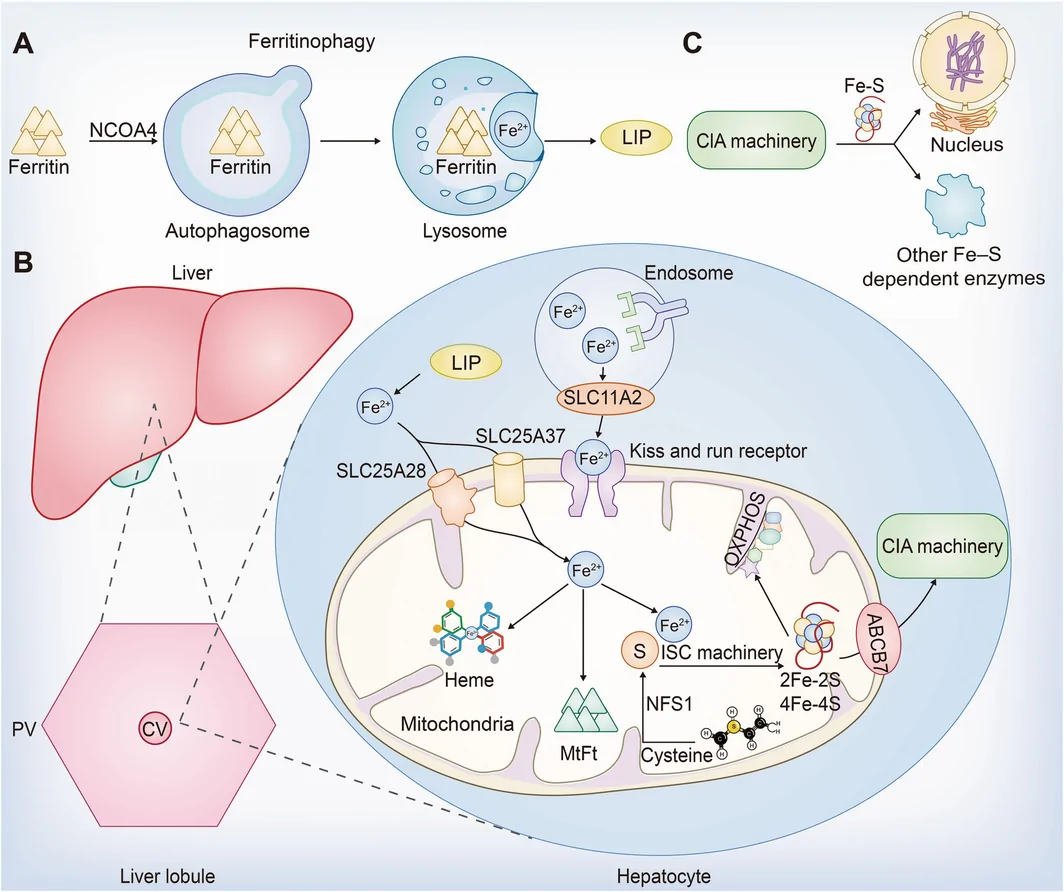
Mobilization and utilization of cellular iron. (A) Iron release via NCOA4‐mediated ferritinophagy during iron deficiency. (B) Mitochondrial iron import for hepatocyte heme and Fe–S cluster biosynthesis. (C) Maturation and delivery of Fe–S clusters via the CIA system. ABCB7, ATP Binding Cassette Subfamily B Member 7; CIA machinery, cytosolic iron–sulphur cluster assembly machinery; CV, central vein; MtFt, mitochondrial ferritin; NCOA4, nuclear receptor coactivator 4; NFS1, NFS1 cysteine desulfurase; OXPHOS, oxidative phosphorylation; PV, portal vein.

Fe–S clusters and heme moieties are directly incorporated into mitochondrial target proteins, particularly components of the electron transport chain (ETC). The ETC transfers electrons from NADH and FADH_2_ to molecular oxygen while generating a transmembrane proton gradient that drives ATP synthesis [53, 54] (Figure 2). Serving as the primary entry point, Complex I utilizes multiple [2Fe–2S] and [4Fe–4S] clusters to facilitate sequential electron relay from NADH to ubiquinone [55, 56, 57]. Operating independently, Complex II accepts electrons from succinate via [2Fe–2S], [3Fe–4S], and [4Fe–4S] clusters for subsequent transfer to ubiquinone [13, 58]. Complex III then uses the Rieske [2Fe–2S] cluster to transfer electrons from reduced ubiquinone to cytochrome *c*. Cytochrome *c* carries a covalently bound *c*‐type heme that cycles between ferrous and ferric states, maintaining protein stability and enabling electron transfer [42, 53]. Finally, Complex IV receives electrons from cytochrome *c* at its Cu_A_ site, passing them through heme a to the heme *a*_3_–Cu_B_ binuclear centre to catalyze the four‐electron reduction of oxygen to water [53, 59]. Thus, mitochondrial iron—in both heme and Fe–S forms—is indispensable for proper ETC assembly and function, making strict iron homeostasis crucial for normal ATP synthesis and sustained organismal bioenergetics.

**FIGURE 2:**
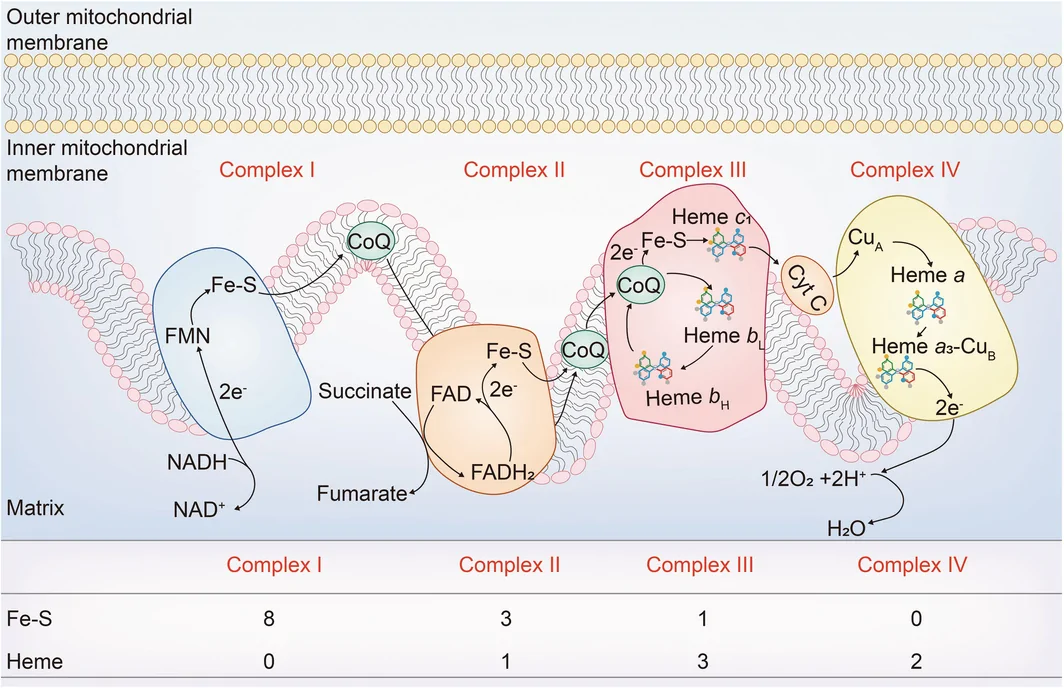
Role of iron in the mitochondrial electron transport chain. CoQ, coenzyme Q; Cu_A_, copper A centre; Cu_B_, copper B centre; Cyt C, cytochrome *c*; FAD, flavin adenine dinucleotide; FADH_2_, Flavin Adenine Dinucleotide (Reduced form); Fe‐S, iron–sulphur cluster; FMN, Flavin MonoNucleotide; NAD^+^, nicotinamide adenine dinucleotide (oxidized form); NADH, nicotinamide adenine dinucleotide (reduced form).

### 2.3 Iron–Sulphur Clusters in Genome Maintenance

In the nucleus, Fe–S clusters are intimately linked to DNA replication. Multiple nuclear enzymes—including DNA primases, polymerases, and glycosylases—rely on these clusters to sustain their catalytic activity [60]. During replication initiation, the DNA polymerase α–primase complex synthesizes the essential RNA–DNA hybrid primer. In budding yeast, the primase regulatory subunit Pri2 harbours an indispensable [4Fe–4S] cluster within its C‐terminal domain to ensure proper primer assembly; loss of this cluster markedly impairs synthesis efficiency [61].

Following primer synthesis, DNA strand elongation at the replication fork is catalyzed by family B DNA polymerases (primarily Pol α, Pol δ, and Pol ε). Structural and biochemical analyses reveal that the C‐terminal catalytic subunits of these polymerases harbour conserved metal‐binding motifs to coordinate [4Fe–4S] clusters [62, 63, 64]. Specifically, Pol2 (the catalytic subunit of Pol ε) contains a conserved [Fe–S] cluster in its C‐terminal domain (CTD). Highlighting its critical role, the *Pol2* ^ΔCTD^ mutant exhibits severely impaired DNA synthesis and fails to fully extend primers [65]. Within the CTD, the cysteine‐rich CysB motif relies on the [4Fe–4S] cluster for proper folding and structural stability. In eukaryotic Pol δ and Pol ζ, this cluster maintains CysB integrity to enable interaction with the regulatory B subunit Pol31; consequently, cluster loss destabilizes Pol δ, severely impairing double‐stranded DNA binding alongside polymerase and exonuclease activities [17, 18, 19, 20].

DNA2, essential for maturing Okazaki fragments during lagging‐strand synthesis, harbours a [4Fe–4S] cluster within its nuclease domain. This cluster stabilizes structural folding, and its disruption markedly reduces nuclease activity [21]. Consequently, perturbing this cluster severely diminishes the nuclease, helicase, and ATPase activities of the enzyme [21, 22].

### 2.4 Iron as a Redox Cofactor in Antioxidant Defence

The iron‐containing heme group serves as the catalytic core for several antioxidant enzymes, notably the highly liver‐expressed catalase. Within its active site, catalase coordinates heme to drive the decomposition of hydrogen peroxide into water and oxygen. This reaction proceeds via a two‐step redox mechanism centred on the heme iron. Upon binding H_2_O_2_, the Fe^3+^ centre is oxidized to an oxoferryl intermediate (Compound I). This intermediate then reacts with a second H_2_O_2_ molecule to regenerate the resting Fe^3+^ state. Through this continuous catalytic cycle, catalase efficiently prevents oxidative damage [23, 24]. Demonstrating this necessity, catalase‐knockout (CKO) mice exhibit elevated hepatic oxidative stress even under normal diets, highlighting the essential role of heme iron in maintaining liver redox homeostasis [25].

### 2.5 Iron in Immune Cell Function and Polarization

Iron centrally regulates the immune system by supporting effector molecule synthesis alongside immune cell proliferation and differentiation. In neutrophils and macrophages, the NADPH oxidase complex contains multiple heme‐ or non‐heme iron‐dependent subunits. By alternating oxidation states, iron facilitates electron transfer to reduce molecular oxygen to superoxide anions (O_2_·^−^). Superoxide then serves as a precursor for other reactive oxygen species (ROS)—including hydrogen peroxide and hydroxyl radicals—essential for pathogen clearance and inflammatory modulation [26, 27].

Similarly, the heme‐containing nitric oxide synthase (NOS) family relies on iron for catalytic activity. The heme iron directly participates in oxygen activation and L‐arginine oxidation to yield nitric oxide (NO) and citrulline. NO subsequently exerts diverse immunological functions, including vasodilation, pathogen inhibition, and immune regulation [28, 29, 30].

In adaptive immunity, iron critically drives T lymphocyte development and proliferation. Specifically, CD4^+^CD25^+^FoxP3^+^ cells can differentiate into induced regulatory T cells (iTregs) [31]. T_H_1 cell proliferation shows a pronounced dependence on iron availability; accordingly, iron chelation markedly suppresses their expansion [32]. Mechanistically, sufficient iron promotes T_H_1 polarization by activating the AKT–mTOR–PFKFB4 pathway to enhance glucose metabolism in CD4^+^ T cells. Conversely, iron chelation downregulates this metabolic axis, reducing proinflammatory T‐cell populations while increasing Treg abundance [33]. During chronic *Salmonella typhimurium* infection, elevated iron further suppresses T_H_1 differentiation and IFN‐γ production by upregulating the inhibitory receptor TIM‐3 [31]. In contrast, T_H_2 cells are considerably less sensitive to iron depletion, allowing low‐iron conditions to preferentially support T_H_2 polarization [32].

CD8^+^ cytotoxic T lymphocytes (CTLs) recognize endogenous antigens presented by MHC class I molecules to eliminate virus‐infected or tumour cells. Iron deficiency or chelation reduces key proinflammatory cytokines (e.g., IL‐2, IFN‐γ, and TNF), thereby limiting CTL proliferative capacity. Ferric supplementation restores normal cell‐cycle progression, potentially by enhancing ribonucleotide reductase activity to support DNA synthesis [66, 67]. Conversely, iron overload downregulates granzyme B and perforin, alters pro‐apoptotic factor balances, and increases CTL susceptibility to ROS‐mediated apoptosis [68].

Iron also robustly regulates innate immunity, particularly macrophage polarization. In response to environmental cues, macrophages differentiate into classically activated (M1) or alternatively activated (M2) phenotypes [69]. Elevated iron enhances the expression of M1 markers, including IL‐6, TNF‐α, and IL‐1β [34]. M1 macrophages accumulate intracellular iron through high hepcidin and ferritin expression coupled with reduced SLC40A1‐mediated export [35]. This retention limits extracellular iron availability, effectively restricting pathogen access. Conversely, M2 macrophages (induced by IL‐4 and IL‐13) acquire iron primarily via CD163‐mediated uptake of heme‐bound iron; consequently, CD163 absence impairs M2 polarization [36, 37]. Unlike M1 cells, M2 macrophages recycle and export internalized iron, maintaining low intracellular levels while increasing extracellular availability. This outward iron flux supports cell proliferation and tissue remodelling, making M2 macrophages critical for wound healing and tumour progression [38, 39].

## 3 Maintenance of Iron Homeostasis

The maintenance of iron homeostasis involves a highly coordinated lifecycle, progressing from intestinal absorption to complex hepatic processing. As the central iron reservoir, the liver utilizes diverse mechanisms—spanning classical and non‐classical pathways in hepatocytes alongside distinct routes in non‐parenchymal cells—to govern iron uptake, internal sequestration, mobilization, and export. To prevent iron dyshomeostasis, this entire metabolic journey is stringently controlled by a dual‐layered regulatory framework. Systemically, the BMP‐SMAD and IL‐6‐STAT3 signalling pathways, coupled with the EPO‐erythroferrone (ERFE) axis, coordinate body‐wide iron distribution. Concurrently, intracellular autonomous regulation is driven by the IRP‐IRE system and hypoxia‐sensing pathways, which dynamically fine‐tune local cellular iron transport and storage. Together, these systemic and cellular mechanisms ensure a robust and precise iron balance.

### 3.1 Intestinal Absorption of Iron

In adults, dietary iron is predominantly absorbed in the duodenum and proximal jejunum [70, 71]. Luminal iron exists in either heme (animal‐derived) or non‐heme (plant‐derived) forms. Non‐heme iron typically presents as ferric iron [72]. These distinct forms are absorbed by enterocytes via divergent molecular mechanisms at the brush‐border membrane (Figure 3).

**FIGURE 3:**
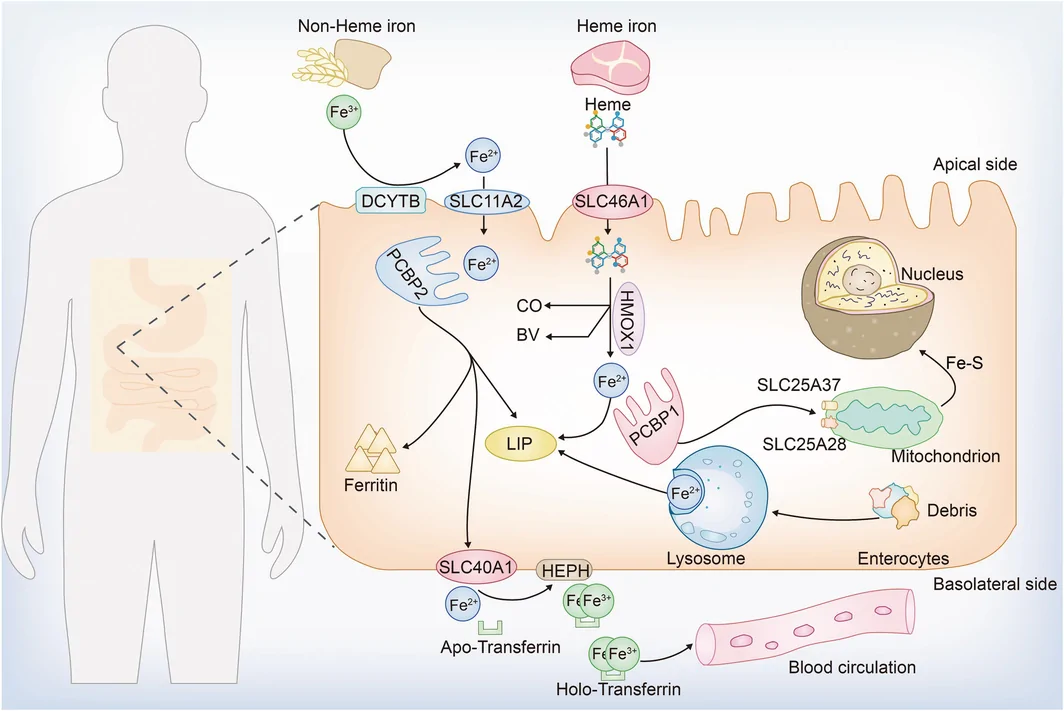
Intestinal absorption and intracellular trafficking of dietary iron. DCYTB, duodenal cytochrome B; HEPH, hephaestin; HMOX1, heme oxygenase 1; LIP, labile iron pool; PCBP1, poly(rC)‐binding protein 1; PCBP2, poly(rC)‐binding protein 2; SLC11A2, divalent metal transporter 1; SLC25A28, Solute Carrier Family 25 Member 28; SLC25A37, Solute Carrier Family 25 Member 37; SLC46A1, Solute Carrier Family 46 Member 1.

As enterocytes strictly uptake Fe^2+^, luminal Fe^3+^ must first be reduced to this bioavailable state—a conversion mediated by membrane‐bound ferric reductases, predominantly duodenal cytochrome b (DCYTB), in an ascorbic acid‐dependent manner [73, 74]. However, *Dcytb* knockout mice do not exhibit overt iron deficiency, suggesting the presence of alternative reductive pathways [75]. Following reduction, Fe^2+^ traverses the apical membrane primarily via the divalent metal transporter SLC11A2 (DMT1) [76, 77]. This proton‐coupled transport strictly relies on the electrochemical gradient maintained by the brush‐border sodium/hydrogen exchanger 3 (NHE3) and the basolateral Na^+^/K^+^‐ATPase [78]. Emphasizing its necessity, intestinal‐specific deletion of *Slc11a2* in mice causes severe anaemia, confirming its indispensable role in iron absorption [79].

Once internalized, the labile iron pool is distributed according to cellular demand by the metallochaperones PCBP1 and PCBP2 [80, 81, 82]. Under low systemic demand, these chaperones route iron for storage within ferritin, the primary intracellular reservoir [81]. Conversely, when demand escalates, iron is exported across the basolateral membrane via SLC40A1 [83, 84]. To facilitate systemic transport, the exported Fe^2+^ is oxidized back to Fe^3+^ by the multicopper ferroxidase hephaestin (HEPH), enabling its binding to circulating apo‐transferrin [85].

Although absorbed more efficiently than its non‐heme counterpart, the precise mechanisms of heme iron uptake remain incompletely elucidated [86]. SLC46A1 (HCP1/PCFT)—initially identified as a proton‐coupled folate transporter—was proposed to mediate apical heme uptake [87]. Similarly, SLC48A1 (HRG‐1) may contribute to this process, though its physiological role in mammals requires further clarification [88]. Once internalized, the heme porphyrin ring is catabolized by heme oxygenase‐1 (HMOX1) to release Fe^2+^, carbon monoxide, and biliverdin [89]. This liberated Fe^2+^ then merges with the intracellular labile iron pool, where it is either sequestered in ferritin, utilized for cellular metabolism, or exported into circulation via SLC40A1 [90].

### 3.2 Classical Iron Uptake Pathways in Hepatocytes

The liver assimilates iron through multiple distinct pathways. Under physiological conditions, the primary route is the classical transferrin receptor (TFRC)‐mediated pathway, whereas a supplementary non‐transferrin‐bound iron (NTBI) uptake mechanism emerges predominantly under pathological conditions (Figure 4). At the core of the classical pathway, TFRC binds to transferrin (Tf)—a high‐affinity iron‐binding glycoprotein encoded by the *TF* gene—forming a critical regulatory axis for systemic iron homeostasis. The vast majority of circulating iron is transported by Tf to tissues with high iron demand, such as the bone marrow, liver, and skeletal muscle [91]. Structurally, Tf is a bilobed glycoprotein containing two specific binding sites, each accommodating a single iron ion [92]. Neonatal *Trf* ^*hpx/hpx*^ mice exhibit severe iron‐deficiency anaemia and perish within approximately 2 weeks unless rescued by red blood cell transfusions or exogenous Tf supplementation. Interestingly, short‐term Tf administration during early life enables survival until weaning, after which these mice remain viable without further injections. However, owing to this profound systemic transferrin deficiency, adult *Trf* ^*hpx/hpx*^ mice paradoxically develop massive iron accumulation in non‐haematopoietic tissues. In the liver, this iron overload is exceptionally severe, reaching levels nearly 100‐fold greater than wild‐type counterparts and 15 to 20 times higher than those in *Hfe*‐related hereditary hemochromatosis models [93].

**FIGURE 4:**
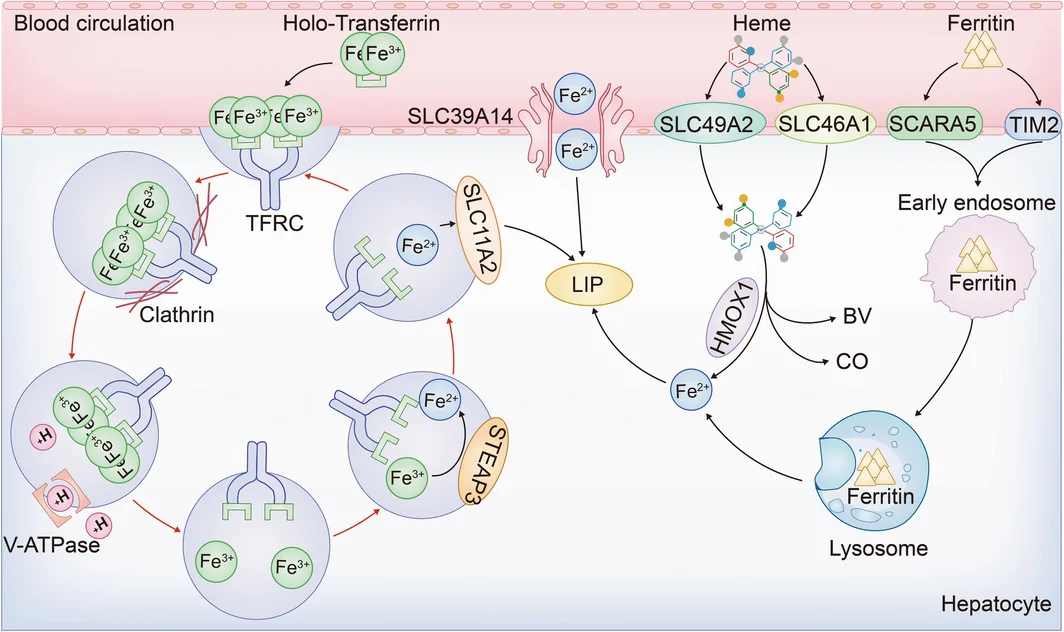
Hepatic iron uptake pathways. FLVCR2, Feline Leukaemia Virus Subgroup C Receptor‐Related Protein 2; SCARA5, Scavenger Receptor Class A Member 5; SLC39A14, Zrt‐ and Irt‐like Protein 14; STEAP3, six‐transmembrane epithelial antigen of prostate 3; TFRC, transferrin receptor; TIM2, T‐cell immunoglobulin and mucin domain 2.

Upon binding Fe^3+^ exported from enterocytes, apo‐transferrin converts into holo‐transferrin (holo‐Tf), which then engages TFRC on target cells to initiate receptor‐mediated endocytosis [94]. Demonstrating the absolute physiological necessity of this receptor, complete *Tfr1* knockout causes embryonic lethality. Conversely, hepatocyte‐specific *Tfr1* knockout mice remain viable and display no overt liver histopathology. Despite this apparent hepatic resilience, these mice exhibit significantly reduced liver LIC, mild hypoferremia, and microcytic anaemia, indicating a profound predisposition to systemic iron‐deficiency anaemia [95].

Following the binding of transferrin to TFRC, the complex undergoes clathrin‐mediated endocytosis—a dynamic process strictly dependent on the cytoskeleton and specific regulatory factors. Initially, the AP‐2 adaptor complex recognizes endocytic motifs within the TFRC cytoplasmic tail to mediate clathrin assembly; correspondingly, knockdown of its μ2 subunit significantly reduces the formation of clathrin‐coated vesicles [96]. Subsequently, dynamin catalyzes vesicle scission, allowing these nascent endosomes to transport iron inward while TFRC is eventually recycled back to the plasma membrane [90, 97, 98]. The precise intracellular targeting of TFRC to early endosomes is orchestrated primarily by Rab GTPases and Rabenosyn‐5. Depleting these factors severely impairs vesicular trafficking: knockdown of *Rab5* reduces both endosome and lysosome numbers [99], whereas Rabenosyn‐5 deficiency profoundly disrupts iron uptake by erroneously misrouting TFRC to lysosomes for degradation [100]. Highlighting the broader systemic importance of this trafficking network, silencing *Rab5* in vivo within the mouse liver triggers severe metabolic disruptions, including hepatomegaly, hypoglycemia, hypercholesterolemia, hyperlipidemia, and marked hepatic glycogen accumulation [101].

In early endosomes, V‐ATPase–driven acidification to ~pH 5.6 reduces holo‐Tf Fe^3+^ affinity, promoting iron release [102, 103]. STEAP3 reduces Fe^3+^ to Fe^2+^, which is then transported into the cytoplasm via SLC11A2 [104]. Although studies have shown that *Slc11a2* ^−/−^ neonatal mice exhibit a threefold increase in hepatic iron content compared to normal levels, liver‐specific *Slc11a2* knockout mice do not display histological abnormalities or evidence of iron metabolism disorders. Non‐heme iron levels in the liver remain within the normal range, even when combined with *Hfe* mutations or a *Trf* ^*hpx/hpx*^ background, indicating that SLC11A2 deletion in hepatocytes does not alter hepatic iron accumulation. Furthermore, in liver‐specific *Slc11a2* knockout models, transferrin‐bound iron (TBI) uptake is reduced by approximately 40%, while NTBI uptake remains unaffected. These findings suggest that SLC11A2 is not an essential pathway for hepatic iron acquisition [105].

Cytosolic Fe^2+^ is shuttled by iron chaperones PCBP1 and PCBP2. PCBP1 coordinates Fe^2+^ as Fe–GSH complexes, delivering it to iron‐dependent targets, including ferritin and non‐heme enzymes [80, 81, 106, 107], and transfers iron to the Glrx3–BolA2 Fe–S cluster chaperone complex via a PCBP1–BolA2 intermediate [108]. Through regulating the availability of cytosolic labile iron, PCBP1 helps maintain the fidelity of the cellular iron‐sensing system and ensures proper iron homeostasis. Embryonic deletion of *Pcbp1* results in embryonic lethality in mice, whereas liver‐specific knockout mice remain viable but display significant hepatic iron dysregulation, characterized by reduced liver iron content and impaired activity of iron‐dependent enzymes [109]. Transcriptomic analysis of *Pcbp1*‐deficient livers revealed abnormal activation of pathways involved in lipid biosynthesis and oxidative stress responses. Moreover, the loss of *Pcbp1* elevates cytosolic labile iron levels, which triggers excessive ROS generation, thereby exacerbating lipid peroxidation and ultimately inducing ferroptosis [82].

Although liver‐specific *Pcbp2* knockout models have not been reported to date, systemic deletion of *Pcbp2* leads to embryonic lethality [109]. PCBP2 functions as an iron chaperone that directly interacts with SLC11A2. It receives Fe^2+^ imported into the cytoplasm and delivers it either to intracellular iron storage proteins or exports it to the extracellular space via SLC40A1. Notably, PCBP1 lacks the ability to deliver Fe^2+^ for cellular export, indicating a distinct role of PCBP2 in intracellular iron homeostasis [110, 111].

### 3.3 Non‐Classical Iron Uptake Pathways in Hepatocytes

Beyond the canonical transferrin receptor pathway, hepatocytes utilize diverse alternative mechanisms to internalize iron. These non‐classical routes primarily encompass the uptake of NTBI, heme, and intact ferritin complexes through specialized membrane transporters and targeted endocytic receptors.

#### 3.3.1 SLC39A14

When plasma iron exceeds the binding capacity of Tf, the classical TFRC‐mediated uptake pathway becomes saturated, resulting in the appearance of non‐transferrin‐bound iron (NTBI) in the circulation [112]. NTBI predominantly exists as loosely chelated complexes with small molecules, such as citrate, and is highly redox‐active, rendering it cytotoxic [113, 114]. The presence of NTBI is closely linked to the pathological progression of iron overload disorders, particularly hereditary hemochromatosis and severe β‐thalassaemia. In these conditions, dysregulated intestinal iron absorption or repeated blood transfusions drive excessive iron deposition in the liver, heart, and pancreas [115, 116].

Among the proposed NTBI transporters, SLC39A8 (ZIP8) and SLC39A14 (ZIP14) are both capable of transporting Fe^2+^. However, SLC39A8 is predominantly expressed in the lung and testis, limiting its relevance to systemic iron handling. In contrast, SLC39A14 is highly expressed in the liver and duodenum—tissues central to whole‐body iron regulation [113]. These expression patterns make SLC39A14 a more plausible mediator of NTBI uptake. Establishing SLC39A14 as the primary mediator of hepatic NTBI clearance, robust functional studies have validated its indispensable role. In mouse models, hepatic overexpression of *Slc39a14* increases iron accumulation, whereas silencing *Slc39a14* significantly reduces iron uptake [117]. Consistently, inhibition of *Slc39a14* in AML12 hepatocytes decreases iron uptake by approximately 30% [118]. Furthermore, *Slc39a14* knockout mice crossed with *Hfe* or *Hfe2* knockout models no longer exhibit the hepatic iron overload characteristic of the single knockouts [119]. This confirms that SLC39A14 constitutes a critical pathway for NTBI uptake in hereditary hemochromatosis, playing a central role in both dietary and genetic iron overload. Consequently, excessive NTBI influx via SLC39A14 directly triggers intracellular iron toxicity and hepatocyte ferroptosis, driving liver pathologies like fibrosis and ischemia–reperfusion injury; conversely, restricting this specific import pathway effectively mitigates such damage [120, 121].

#### 3.3.2 SLC46A1

SLC46A1, also known as proton‐coupled folate transporter (PCFT) or heme carrier protein 1 (HCP1), is a proton‐coupled transporter comprising 12 transmembrane helices [122]. It was initially characterized in intestinal epithelial cells as a mediator of dietary heme iron uptake [123]. However, subsequent studies demonstrated that its primary physiological role is as a high‐affinity folate transporter [124]. In animal models, global deletion of *Slc46a1* causes severe folate deficiency and postnatal lethality. In contrast, liver‐specific knockout mice remain viable but display reduced hepatic iron stores, elevated serum iron, and dysregulated expression of hepcidin and iron transporters. These findings highlight the dual role of SLC46A1 in folate absorption and the maintenance of systemic iron homeostasis [122, 125].

#### 3.3.3 SLC49A2

SLC49A2 (FLVCR2) is a canonical member of the major facilitator superfamily (MFS) of transmembrane transporters. It is widely expressed in the liver, brain, and haematopoietic tissues, including fetal liver and bone marrow. SLC49A2 was initially proposed to function as a heme importer. In TE671 cells, siRNA‐mediated knockdown of *SLC49A2* reduced uptake of the heme analogue ZnMP by 34% and 25%, respectively. This supports a role for SLC49A2 in heme transport [126]. SLC49A2 has also been shown to bind heme‐conjugated agarose, with free heme competing for this interaction. Furthermore, mammalian cells and *Xenopus* oocytes expressing SLC49A2 exhibit enhanced heme uptake [126]. Global deletion of *Slc49a2* in mice results in embryonic lethality, accompanied by abnormal brain vascular development, hydrocephalus, and hypoxic neuronal injury. These manifestations recapitulate key features of human Fowler syndrome [127, 128].

#### 3.3.4 Endocytic Uptake of Ferritin

Ferritin is a spherical protein that serves as a central mediator of iron storage and detoxification. It is composed of two subunit types: H (heavy) and L (light) chains. The H subunit exhibits ferroxidase activity, catalyzing the oxidation of Fe^2+^ to Fe^3+^. In contrast, the L subunit promotes iron nucleation and storage within the central cavity of the ferritin shell. Ferritin typically assembles into a hollow 24‐mer capable of accommodating up to 4500 iron ions [129]. Circulating ferritin concentrations increase under conditions such as inflammation, liver disease, and chronic illness. Therefore, serum ferritin serves as both a marker of systemic iron stores and an acute‐phase reactant [130].

Studies on ferritin uptake are limited. TIM2 has been identified as a receptor for H‐ferritin [131]. Additionally, SCARA5 mediates L‐ferritin uptake in proximal tubular epithelial cells of the kidney [132]. Whether TIM2 and SCARA5 act as specific ferritin receptors in hepatocytes remains to be determined.

### 3.4 Iron Import in Non‐Parenchymal Cells

Beyond hepatocytes, non‐parenchymal cells—particularly macrophages—play an indispensable role in maintaining systemic iron homeostasis through highly specialized scavenging systems. To recycle iron and mitigate oxidative toxicity during hemolytic stress, these cells orchestrate a comprehensive defence network, prominently featuring the sequential activation of the CD163 and CD91 receptor pathways to neutralize cell‐free haemoglobin and heme.

#### 3.4.1 CD163

During intravascular hemolysis, the body initiates its primary defence mechanism. Free haemoglobin (Hb) is rapidly captured by haptoglobin (Hp) to form a stable Hb‐Hp complex. This binding prevents renal filtration loss and mitigates oxidative toxicity. The complex is subsequently recognized and endocytosed by the scavenger receptor CD163, which is specifically expressed on macrophages, such as hepatic Kupffer cells [133, 134]. Upon internalization, the complex is degraded within lysosomes to release heme. Heme oxygenase‐1 (HMOX1) then catalyzes the cleavage of heme into free ferrous ions. These iron ions are either safely sequestered within ferritin or exported into the systemic circulation via SLC40A1. This coordinated process facilitates systemic iron recycling [135].

#### 3.4.2 CD91

During severe or persistent hemolysis, Hp becomes depleted. Consequently, free heme—a molecule with potent lipid peroxidation toxicity—is directly exposed to the circulation, triggering a secondary defence mechanism [136]. Hemopexin (Hpx) binds this free heme with exceptionally high affinity to form a Heme‐Hpx complex. This complex is predominantly recognized and internalized by the receptor CD91 (also known as LRP1), which is expressed on the surfaces of both hepatocytes and macrophages [137]. Following its intracellular release, heme is similarly processed by the HMOX1 catalytic network to liberate iron ions. Concurrently, a portion of the intact Hpx is recycled to the extracellular space for continuous use. Together, these two temporally complementary and highly specific pathways establish a comprehensive defence network. This network effectively neutralizes hemolytic stress and preserves systemic iron homeostasis [138].

### 3.5 Hepatic Iron Sequestration and Mobilization

After Fe^2+^ enters the cytoplasm from endosomes via SLC11A2, it initially accumulates in the LIP. This pool serves as a dynamic, low‐molecular‐weight, exchangeable iron reservoir composed mainly of iron‐glutathione and iron‐citrate complexes [139, 140]. When cellular iron is scarce or metabolic demand rises, iron stored in ferritin can be rapidly mobilized. Early studies using ^125^I‐labelled [^59^Fe] ferritin in hepatocytes indicated that ferritin is trafficked to lysosomes and mitochondria. Within the lysosomes, degradation releases this stored iron. A portion of the released iron is recycled into cytosolic ferritin, while the remainder is incorporated into mitochondria [141].

Recent work has established that ferritin mobilization occurs predominantly through ferritinophagy, a selective autophagic process mediated by NCOA4 [142, 143]. NCOA4 acts as an autophagy receptor, recognizing ferritin—particularly the heavy chain FTH1—and delivering it to autophagosomes [144]. Key autophagy proteins such as LC3 (MAP1LC3B) facilitate ferritin encapsulation and trafficking [145, 146]. Following autophagosome‐lysosome fusion, ferritin is degraded by lysosomal proteases. Subsequently, the liberated Fe^3+^ is reduced to bioavailable Fe^2+^ by intracellular reductive systems. Fe^2+^ is then exported from lysosomes to the cytoplasm via SLC11A2 or MCOLN1 (TRPML1). This export efficiently replenishes the LIP [147].

In hepatocytes, ferritinophagy is critical for maintaining iron homeostasis. Loss or dysfunction of NCOA4 leads to ferritin accumulation and impaired iron release. Global *Ncoa4*‐knockout mice exhibit elevated hepatic and splenic iron compared with wild‐type animals [148]. Liver‐specific *Ncoa4* deletion causes decreased serum transferrin saturation, anaemia, downregulation of hepcidin, and increased markers of hepatic inflammation and mild fibrosis. When subjected to a high‐iron diet for 2 months, *Ncoa4*‐deficient mice display elevated ALT and AST levels and more pronounced hepatic steatosis than wild‐type controls. Consequently, liver‐specific *Ncoa4* knockout reduces lifespan by ~15%. This highlights the role of hepatic ferritinophagy in modulating iron‐dependent liver disease progression [149].

### 3.6 Iron Export Pathways in Hepatocytes

The primary pathway involves the efflux of ferrous iron via SLC40A1, followed by oxidation to ferric iron by ceruloplasmin (CP) for binding to transferrin. Beyond this classic route, cells also eliminate iron in the form of ferritin through non‐canonical autophagy and exosome‐mediated secretion. Additionally, iron is exported as heme through specific transporters like FLVCR1a, MRP5, and ABCG2 to ensure safe systemic transport and recycling. Unlike the hepcidin‐regulated SLC40A1 pathway, the control mechanisms for these non‐classic routes remain largely unknown (Figure 5).

**FIGURE 5:**
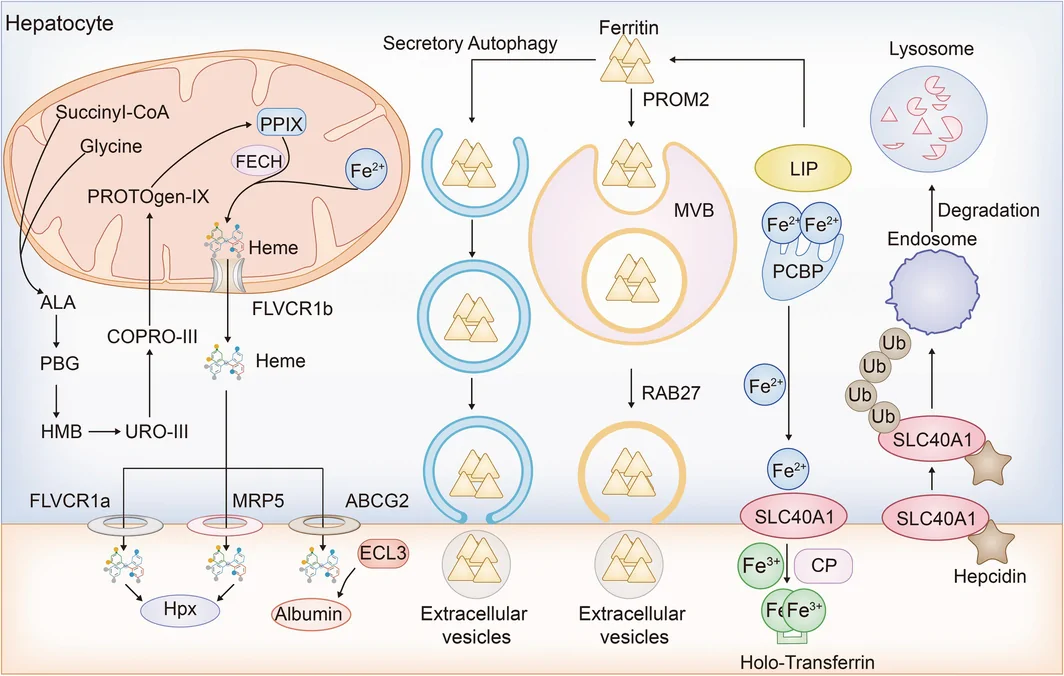
Iron export mechanisms in hepatocytes. ABCG2, ATP‐Binding Cassette Subfamily G Member 2; ALA, 5‐aminolevulinic acid; Albumin, serum albumin; COPRO‐III, coproporphyrinogen III; CP, ceruloplasmin; ECL3, extracellular loop 3; Fe^2+^, ferrous iron; Fe^3+^, ferric iron; FECH, ferrochelatase; FLVCR1a, Feline Leukaemia Virus Subgroup C Receptor 1a; FLVCR1b, Feline Leukaemia Virus Subgroup C Receptor 1b; HMB, hydroxymethylbilane; Holo‐Transferrin, transferrin bound with Fe^3+^; Hpx, hemopexin; LIP, labile iron pool; MRP5, multidrug resistance‐associated protein 5; MVB, multivesicular body; PBG, porphobilinogen; PCBP, poly(rC)‐binding protein; PPIX, protoporphyrin IX; PROM2, prominin‐2; PROTOgen‐IX, protoporphyrinogen IX; RAB27, RAS‐related protein Rab‐27; SLC40A1, Solute Carrier Family 40 Member 1; Succinyl‐CoA, succinyl coenzyme A; URO‐III, uroporphyrinogen III.

#### 3.6.1 SLC40A1

At the cellular level, SLC40A1 actively exports intracellular Fe^2+^ into the circulation [84]. Ceruloplasmin (CP) then oxidizes the exported Fe^2+^ to Fe^3+^, enabling its subsequent binding to transferrin (Tf) to form holo‐Tf for systemic transport [71]. In the liver, SLC40A1 is highly expressed in hepatocytes and Kupffer cells. Hepatocyte‐specific *Slc40a1* knockout mice exhibit hepatic iron accumulation, reduced serum iron levels, and decreased transferrin saturation. By contrast, global *Slc40a1* deletion results in severe iron sequestration, systemic anaemia, and embryonic lethality [150]. Notably, liver‐specific deletion of *Slc40a1* can induce hepatic iron deposition and, in some mice, early features of liver fibrosis. These findings highlight the critical role of SLC40A1 in maintaining hepatic iron homeostasis and preventing iron‐induced liver injury [151].

#### 3.6.2 Iron Trafficking via Exosomes and Non‐Canonical Autophagy

In addition to classical iron efflux via SLC40A1, hepatocytes can release iron into the extracellular space in the form of ferritin through non‐canonical pathways, including autophagy and exosome‐mediated secretion [152]. The absence of glycosylation on circulating ferritin supports the involvement of a non‐classical, autophagy‐dependent mechanism [153].

Exosomal ferritin release represents another important pathway. Specifically, Prominin‐2 promotes multivesicular body (MVB) formation and enhances the secretion of ferritin‐containing exosomes. This process facilitates iron export and protects cells from ferroptosis [154]. Supporting this mechanism, ferritin has been detected in exosomes isolated from human urine. This finding provides direct evidence for MVB/exosome‐mediated ferritin export [155].

#### 3.6.3 Heme‐Mediated Iron Export

In addition to classical iron export via ferrous ions or ferritin, cells eliminate iron in the form of heme, a lipophilic and potentially cytotoxic molecule. Major heme exporters include FLVCR1a, MRP5, and ABCG2. FLVCR1a mediates high‐affinity heme efflux at the plasma membrane. Its loss leads to hepatic heme and iron accumulation, oxidative stress, and disruption of cytochrome P450 function [156, 157]. MRP5 exports heme and porphyrins. Genetic knockdown in model organisms impairs inter‐tissue heme transport and hemoglobinization [158]. Meanwhile, ABCG2 facilitates heme and protoporphyrin IX export. It uses its extracellular loop 3 (ECL3) to transfer heme to extracellular albumin, thereby minimizing free heme toxicity [159]. Once in the circulation, heme is rapidly scavenged by Hpx, which mediates hepatic uptake and iron recycling. Concurrently, albumin acts as a lower‐affinity, transient transporter following ABCG2‐mediated export. Together, these coordinated pathways ensure safe heme export, systemic distribution, and iron homeostasis.

### 3.7 Regulation of Iron Homeostasis

Systemic iron homeostasis is synergistically maintained by the small intestine, liver, and reticuloendothelial system. At the systemic level, hepcidin synthesis is dynamically governed by the BMP/SMAD, IL‐6‐STAT3, and EPO‐ERFE pathways in response to iron fluctuations, inflammation, and erythropoietic demands, respectively. Concurrently, intracellular iron balance is subjected to post‐transcriptional management via the IRP‐IRE and HIF‐2α regulatory systems. Ultimately, this multi‐tiered regulatory network, spanning from systemic to cellular levels, collectively ensures dynamic iron homeostasis under complex physiological conditions, including ischemia and hypoxia (Figure 6).

**FIGURE 6:**
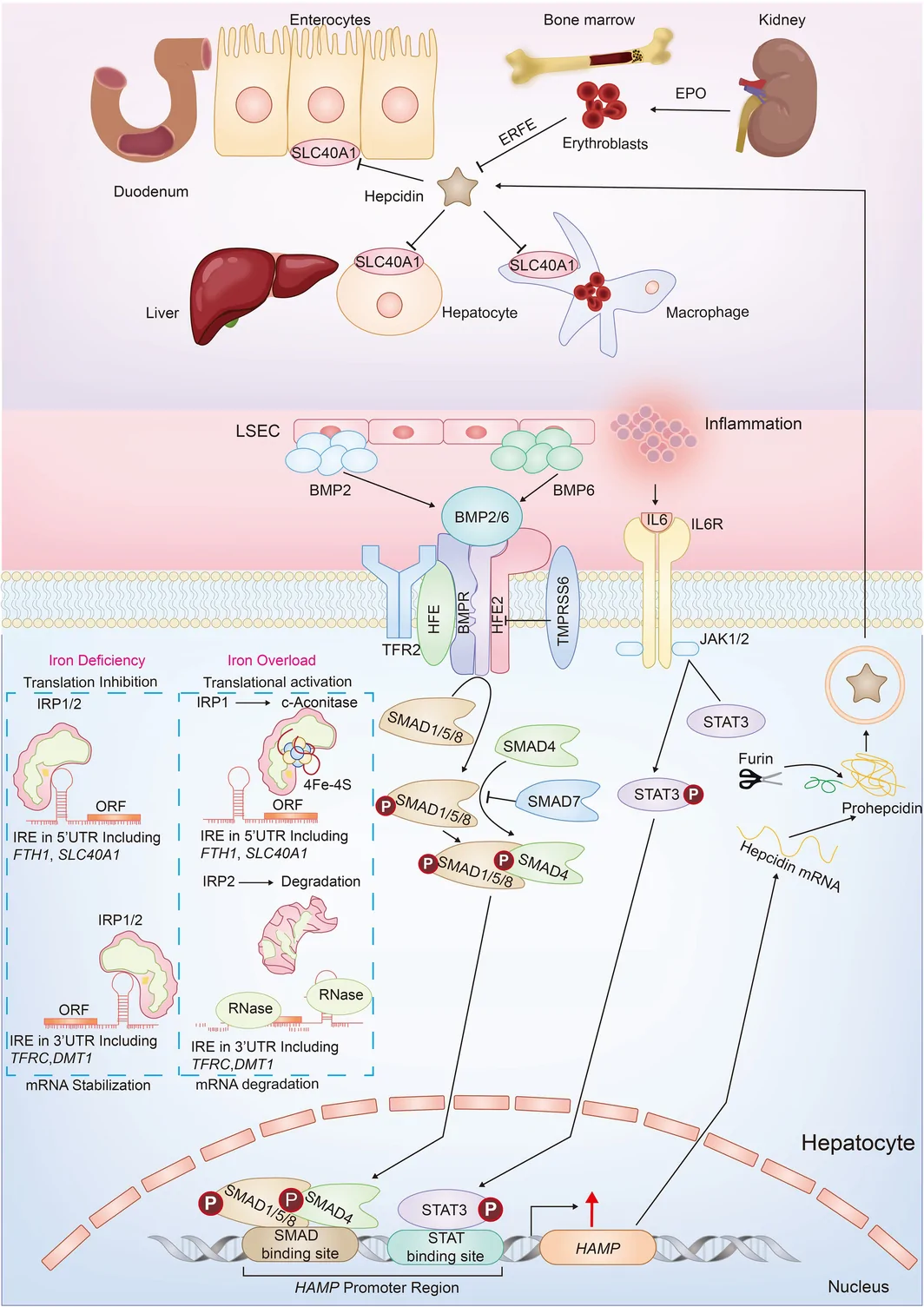
Regulation of iron homeostasis. 3′UTR, 3′ untranslated region; 5′UTR, 5′ untranslated region; BMP2, bone morphogenetic protein 2; BMP6, bone morphogenetic protein 6; BMPR, bone morphogenetic protein receptor; EPO, erythropoietin; FTH1, ferritin heavy chain 1; IL6, interleukin 6; IL6R, interleukin 6 receptor; IRE, iron responsive element; IRP1, iron regulatory protein 1; IRP2, iron regulatory protein 2; JAK1/2, Janus kinase 1/2; LSEC, liver sinusoidal endothelial cell; ORF, open reading frame; SMAD1/5/8, SMAD family member 1/5/8; SMAD4, SMAD family member 4; SMAD7, SMAD family member 7; STAT3, signal transducer and activator of transcription 3; TMPRSS6, transmembrane serine protease 6.

#### 3.7.1 Systemic Iron Regulation

At the core of systemic iron regulation lies the BMP‐SMAD signalling axis, which serves as the primary sensor for physiological iron fluctuations. During iron overload, liver sinusoidal endothelial cells (LSECs) act as central sensors, upregulating *Bmp6* expression through multiple intricate pathways. Specifically, excess iron triggers mitochondrial ROS accumulation to activate the Nrf2 transcription factor [160]. Meanwhile, an independent iron‐triggered signalling pathway initiates MAPK cascades to activate ETS1. Together, these processes synergistically drive *Bmp6* transcription [161]. Furthermore, iron overload suppresses mTORC2 activity, leading to the nuclear translocation of the transcription factor FoxO1. This factor then directly activates the promoters of *Bmp6* and *Bmp2* [162]. The secreted BMP2/BMP6 heterodimers bind via paracrine signalling to a hepatocyte receptor complex comprising HFE, TFR2, and the coreceptor HJV [163, 164]. This activates ALK receptor kinases, leading to the phosphorylation of SMAD1/5/8. These phosphorylated SMADs then associate with SMAD4 and translocate to the nucleus to robustly induce hepcidin (*HAMP*) transcription [165]. To prevent signal hyperactivation, the transmembrane protease TMPRSS6 specifically cleaves cell‐surface HJV to disrupt receptor integrity [166]. Concurrently, newly synthesized SMAD7 mediates the ubiquitination and degradation of the receptor complex [167]. During iron deficiency, this positive sensing network is silenced. Active mTORC2 promotes the phosphorylation and nuclear exclusion of FoxO1, resulting in a precipitous decline in hepcidin.

Operating in parallel to this iron‐sensing network, the IL‐6‐STAT3 signalling axis mediates urgent iron redistribution during inflammatory stress [168]. Upon infection or iron‐induced tissue injury, macrophages release substantial amounts of the proinflammatory cytokine IL‐6. Circulating IL‐6 binds to the IL‐6R/gp130 complex on hepatocytes. This interaction rapidly activates intracellular JAK2 kinases. Activated JAK2 induces the phosphorylation, dimerization, and nuclear translocation of STAT3. Once in the nucleus, STAT3 robustly drives hepcidin expression independently of systemic iron levels [169]. Elevated hepcidin forces macrophages to internalize and degrade SLC40A1, sequestering iron intracellularly to limit pathogen access [170]. When inflammation resolves, the upregulation of intracellular negative regulators (e.g., SOCS3) terminates JAK2‐STAT3 signalling. Consequently, the body can resume normal iron mobilization [171].

Additionally, systemic iron homeostasis is strictly coordinated with erythropoietic demand via the EPO‐ERFE pathway. This pathway constitutes a potent negative feedback loop from the haematopoietic system to hepatic iron metabolism. Blood loss or hypoxia induces substantial renal EPO secretion. This hormone strongly stimulates bone marrow erythroid progenitor proliferation and triggers their explosive secretion of ERFE [172]. Functioning as an endocrine hormone, ERFE reaches the liver to directly bind and sequester BMP ligands (primarily BMP6) within the microenvironment. This sequestration effectively blunts SMAD signalling in hepatocytes [163]. This causes a dramatic suppression of hepcidin expression. Consequently, iron export channels in the intestine and reticuloendothelial system open fully to meet the demands of erythropoiesis [173].

Interestingly, the mouse HAMP homologues, *Hamp* and *Hamp2*, are specifically expressed in Zone 2 of the liver lobule [174], which serves as a vital hub for hepatocyte homeostasis [175, 176, 177]. While it is established that iron overload induces Cyclin D1 and disrupts the cell cycle [178], the question of how iron homeostasis regulates hepatocyte repopulation during normal steady‐state conditions has yet to be answered.

#### 3.7.2 Intracellular Iron Regulation

Beyond systemic hormonal control, individual cells are equipped with intrinsic mechanisms to manage local iron availability. Foremost among these is the IRP‐IRE network. This network constitutes an autonomous iron‐balancing system operating entirely at the post‐transcriptional level [179]. During iron deficiency, cytosolic IRP1 (lacking an iron–sulphur cluster) and stabilized IRP2 expose high‐affinity RNA‐binding domains. They bind to IREs at the 3′ UTR of target mRNAs (e.g., *TFRC*, *SLC11A2*) to prevent degradation and enhance iron import [180]. Conversely, binding to the 5′ UTR (e.g., *SLC40A1*, *FTH1*, *FTL*) creates steric hindrance that blocks translation. This steric hindrance suppresses iron export and storage [181]. In iron‐replete cells, IRP1 assembles an iron–sulphur [4Fe‐4S] cluster and functions as a cytosolic aconitase. This enzyme catalyzes the stereospecific conversion of citrate to isocitrate via the intermediate *cis*‐aconitate [182]. In this ‘closed’ conformation, the IRE‐binding site of IRP1 is inaccessible, and IRP2 is targeted for proteasomal degradation [183, 184, 185]. Thus, the translation of the *SLC40A1* and *FTH1* transcripts proceeds unhindered, resolving intracellular iron toxicity.

Complementing this post‐transcriptional regulation, specific transcriptional responses are mobilized to enhance cellular iron management under stress. Specifically, by integrating hypoxia and iron‐deficiency signals, HIF‐2α coordinates hypoxia‐induced erythropoietic signalling and systemic iron mobilization. It does this partly through the induction of extra‐renal EPO production in the liver under severe stress [186]. This mechanism is governed by prolyl hydroxylases (PHDs), whose ability to target HIF‐2α for degradation strictly depends on both oxygen and ferrous iron as co‐substrates. During hypoxia or iron deficiency, PHDs are rapidly inactivated, allowing HIF‐2α to escape VHL‐mediated proteasomal degradation [187]. The accumulated HIF‐2α translocates to the nucleus and binds to hypoxia‐response elements (HREs). This binding robustly upregulates the transcription of classical target genes essential for iron transport and utilization (such as *SLC11A2*, *SLC40A1*, and *TFRC*) [188]. Concurrently, this hypoxic adaptation facilitates intracellular iron recycling mechanisms, including NCOA4‐mediated ferritinophagy, to meet elevated erythropoietic demands [189]. Ultimately, this precise transcriptional network allows the local hepatic microenvironment to directly couple oxygen sensing with systemic iron mobilization and erythropoiesis.

#### 3.7.3 Epigenetic Regulation of Iron

Beyond classical regulatory systems, recent studies reveal that iron homeostasis is also tightly governed by epigenetic networks. This emerging layer of control precisely orchestrates the dynamic balance of iron import, storage, and export.

In the transferrin‐bound iron uptake pathway, the translation of *TFRC* is enhanced by m^6^A RNA methylation reader proteins, such as YTHDF1. Concurrently, specific endocrine stimuli can induce the epigenetic silencing of the *TFRC* promoter, thereby downregulating iron uptake [190, 191]. In the endosomal iron release pathway, the expression of the metalloreductase *STEAP3* is governed by the remodelling of its promoter methylation status, which is mediated by methylation reader proteins like MBD2 [192]. Additionally, the transcriptional activity of the transmembrane import channel *SLC11A2* is regulated by DNA methylation. Specifically, differential DNA methylation of the *SLC11A2* gene has been closely linked to altered iron uptake and subsequent iron overload [193]. Furthermore, the transporter *SLC39A14*, which is responsible for non‐transferrin‐bound iron uptake, also relies on promoter hypomethylation to overcome transcriptional inhibition. Interestingly, the metal ions imported by SLC39A14 can act as intracellular cofactors. These cofactors help maintain the catalytic activity of histone deacetylases (HDACs) [194, 195].

Regarding iron storage, intracellular labile iron is primarily sequestered within ferritin complexes, which consist of heavy (FTH1) and light (FTL) chains. Changes in environmental signals can induce the epigenetic silencing of the *FTH1* gene. This silencing reduces the synthesis of ferritin complexes, thereby dynamically adjusting the cellular iron storage capacity [190].

In the context of iron export and systemic regulation, the expression of the cell membrane iron efflux pump *SLC40A1* is co‐regulated by DNA methylation and chromatin remodelling. Specifically, the DNA methyltransferase DNMT3B mediates the hypermethylation of the *SLC40A1* promoter to repress its transcription. Furthermore, reduced activity of histone demethylases can shift the chromatin at distal regulatory regions of *SLC40A1* into a closed state. This conformational change physically blocks the binding of the transcriptional machinery, leading to the downregulation of *SLC40A1* [196, 197]. Systemically, the transcription of *HAMP* is directly activated by the hypomethylation of its promoter [198]. Collectively, the epigenetic regulation of these transmembrane transporters and macromolecular hormones plays a critical role in maintaining iron homeostasis at both the cellular and systemic levels.

## 4 Clinical Diagnosis and Iron‐Related Metabolic Disorders

Since the 1996 discovery of the *HFE* gene mutation (C282Y)—the primary driver of hereditary hemochromatosis [199]—iron‐related liver disorders have garnered intense scientific scrutiny.

### 4.1 Diagnosis of Iron Overload

Historically, liver biopsy was the gold standard for diagnosing hemochromatosis (HC) and quantifying liver LIC. However, its invasiveness and cost have driven a shift toward non‐invasive diagnostic models. Diagnosis begins with haematological screening [200]. HC is suspected when transferrin saturation (TSAT) exceeds 45% and serum ferritin is > 200 μg/L in premenopausal women. For men and postmenopausal women, the thresholds are TSAT > 50% and ferritin > 300 μg/L [201]. Positive screens are then confirmed via *HFE* and non‐*HFE* genetic testing. Early diagnosis enables timely phlebotomy. This prevents irreversible organ damage and restores normal life expectancy.

To assess tissue iron burden, magnetic resonance imaging (MRI) has largely replaced liver biopsy. Confounder‐corrected R_2_* relaxometry is now the preferred evidence‐based approach. MRI scanners convert tissue relaxation rates directly into absolute LIC values. This non‐invasive quantification guides clinical management. LIC < 1.8 mg/g (32 μmol/g) dry weight is normal. Levels between 7.0 and 15.0 mg/g (125–269 μmol/g) indicate moderate iron overload with rising complication risks. Concentrations > 15.0 mg/g (269 μmol/g) signify severe overload and an elevated risk of early mortality [202, 203].

Despite these advancements, significant diagnostic challenges persist. First, serum ferritin lacks diagnostic specificity [204]. As an acute‐phase reactant, it frequently elevates during systemic inflammation, alcohol consumption, or MASLD. This heavily confounds the identification of true iron overload [205]. Second, widespread MRI implementation is hindered by high costs and limited access to specialized calibration software in resource‐limited settings. Furthermore, concurrent severe hepatic steatosis can cause significant signal interference, complicating accurate iron quantification [202, 206, 207].

### 4.2 Hereditary Hemochromatosis

Hereditary hemochromatosis (HH) encompasses a group of autosomal genetic disorders characterized by dysregulated iron metabolism, which drives excessive dietary iron absorption and progressive iron deposition across multiple tissues, particularly the liver [208, 209]. Hallmark clinical features include elevated serum iron, increased transferrin saturation, and parenchymal iron accumulation. HH is traditionally classified into four major types. Type 1, the most common variant, is primarily associated with *HFE* mutations (notably C282Y and H63D). Type 2 (juvenile hemochromatosis) presents at an earlier age and results from mutations in *HFE2* (hemojuvelin, HJV) or *HAMP*. Type 3 arises from mutations in *TFR2*. Mechanistically, TFR2 and HFE2 cooperatively regulate the BMP–SMAD signalling pathway to promote hepcidin expression. Consequently, *TFR2* mutations impair hepcidin synthesis and exacerbate systemic iron overload. Finally, Type 4 (ferroportin disease) is caused by *SLC40A1* mutations and follows an autosomal dominant inheritance pattern [200, 203]. Detailed classifications are summarized in Table 2.

**TABLE 2:** Classification of patients with hereditary hemochromatosis.

Classification	Gene	Protein	Function	Inheritance	Main clinical features	Severity
Type 1 (Classic HH)	*HFE*(C282Y)	HFE	Regulates hepcidin expression in response to iron	AR	Onset in 4th–5th decade. Fatigue, arthralgia, elevated TSAT and ferritin, liver disease, diabetes, bronze skin.	Variable
	*HFE*(H63D)	HFE	Regulates hepcidin expression in response to iron	AR	Milder phenotype, often compound heterozygous with C282Y.	Variable
Type 2 (Juvenile HH)	*HFE2*	Haemojuvelin	Coreceptor for BMP signalling to upregulate *HAMP*	AR	Onset before 30. Severe cardiomyopathy and hypogonadism.	Severe
	*HAMP*	Hepcidin	Binds ferroportin to inhibit cellular iron export	AR	Onset before 30. Severe cardiomyopathy and hypogonadism.	Severe
Type 3	*TFR2*	TFR2	Senses holo‐transferrin to regulate hepcidin	AR	Onset in the 4th–5th decade with liver fibrosis or cirrhosis.	Variable
Type 4 (Ferroportin disease)	*SLC40A1*	Ferroportin	Cellular iron exporter	AD	Anaemia with reduced tolerance to phlebotomy.	Mild

Abbreviations: AD, autosomal dominant; AR, autosomal recessive; HAMP, hepcidin antimicrobial peptide; HFE, human homeostatic iron regulator protein; HFE2, homeostatic iron regulator 2; HH, hereditary hemochromatosis; SLC40A1, Solute Carrier Family 40 Member 1; TFR2, transferrin receptor 2.

In HH patients, iron accumulates predominantly in hepatocytes, elevating oxidative stress and activating hepatic stellate cells to drive liver fibrosis progression. To investigate this pathogenesis, *Hfe* knockout (*Hfe* ^−/−^) mice are classically employed as the standard experimental model. However, accurately modelling this initial iron loading in these rodents is confounded by species‐specific absorption dynamics; unlike humans, who rely entirely on dietary vitamin C to reduce Fe^3+^ for SLC11A2‐mediated uptake, mice endogenously synthesize ascorbate via the *Gulo* gene [210]. Despite these fundamental model differences, hepatocellular iron deposition in humans correlates with an approximately 1.7‐fold increased risk of fibrosis beyond stage 1 [211]. Left untreated, this fibrosis frequently progresses to cirrhosis [209]. Up to 9% of male patients with *HFE* hemochromatosis and approximately 2% of affected women develop cirrhosis [212]. Consequently, the risk of HCC is markedly elevated in HH, with studies reporting a 100‐ to 200‐fold increase compared to the general population [213]. Crucially, bridging the translational gap for these late‐stage outcomes faces significant experimental limitations. Standard *Hfe* ^*−/−*^ mice rarely develop spontaneous cirrhosis or HCC [214]. They typically require secondary chemical insults or genetic ‘multi‐hits’ to mimic the advanced pathogenesis of human liver disease [215].

### 4.3 Iron and MASLD

Hepatic iron overload is frequently observed in MASLD patients [216]. Accumulating evidence indicates that this overload directly contributes to hepatic steatosis. Specifically, overexpression of iron uptake genes—such as *Slc11a* and *Tfrc*—increases intracellular iron in hepatocytes, driving MASLD initiation and progression [217]. Abnormal iron accumulation impairs mitochondrial function and facilitates lipid deposition within these cells [218, 219]. In vitro studies further demonstrate that iron loading upregulates key lipogenic genes, notably *Fasn* (fatty acid synthase) and *Srebp* (sterol regulatory element‐binding proteins), thereby enhancing *de novo* lipogenesis and lipid accumulation [220]. Supporting these findings, chronic iron dextran administration in animal models induces hepatic iron overload, increases lipid deposition, and exacerbates hepatocellular steatosis [218]. Furthermore, in MASLD, excess iron catalyzes the Fenton reaction to generate ROS; this triggers oxidative stress, promotes lipid peroxidation, and induces hepatocellular injury, ultimately accelerating disease progression [221]. These experimental models effectively demonstrate iron‐induced oxidative damage. However, parenteral iron injections bypass physiological intestinal absorption. This artificial route predominantly loads macrophages instead of hepatocytes, creating a significant translational gap in modelling diet‐driven human MASLD [222, 223].

Beyond hepatocytes, excess iron activates Kupffer cells and other hepatic macrophages, prompting the secretion of inflammatory cytokines like IL‐6 and TNF‐α [224]. Elevated IL‐6 then stimulates the expression of hepcidin (HAMP), a hormone frequently upregulated in MASLD patients [225, 226]. Interestingly, despite this elevated hepcidin, iron predominantly accumulates in the liver during MASLD. This paradox highlights a profound disruption in iron homeostasis, though the precise molecular mechanisms remain to be fully elucidated.

### 4.4 Iron and Viral Hepatitis

Evidence indicates that iron overload during hepatitis B virus (HBV) and hepatitis C virus (HCV) infections correlates with poorer prognosis and accelerated liver disease progression. Approximately 40% of chronic hepatitis B (CHB) patients exhibit elevated hepatic iron, with serum iron and ferritin frequently exceeding normal ranges [227, 228].

Regarding HCV—an enveloped, positive‐sense single‐stranded RNA virus [229]—iron overload complicates 10%–42% of chronic hepatitis C (CHC) cases, with 20%–35% of patients displaying elevated serum iron, ferritin, and transferrin saturation [230]. Mechanistically, this disrupted iron metabolism is linked to decreased *HAMP* expression and increased *TFRC* levels [231, 232], which cooperatively promote iron accumulation. Consequently, this overload may further enhance viral replication and exacerbate disease progression [225]. Genetically, *HFE* mutations—particularly the C282Y variant in either homozygous or heterozygous states—predispose HCV‐infected individuals to increased hepatic iron, directly contributing to steatosis and liver fibrosis [233, 234]. Corroborating these clinical observations, HCV‐infected mouse models with iron overload exhibit aggravated hepatic steatosis and a markedly increased risk of hepatocarcinogenesis [213]. However, translating these preclinical findings is hindered by strict species differences and model limitations [235]. Lacking human‐specific viral entry receptors, wild‐type mice are naturally resistant to HCV, forcing these studies to rely on highly manipulated transgenic or chimeric models that fail to fully recapitulate the complex immune‐iron crosstalk seen in human chronic infection [236].

### 4.5 Iron and Liver Fibrosis and Cirrhosis

In both clinical settings and rodent models of hepatic fibrosis and cirrhosis, iron overload is consistently associated with poor disease outcomes. Hepatic fibrosis represents a pathological repair response to persistent liver injury, characterized by excessive extracellular matrix (ECM) accumulation. This progressive ECM deposition disrupts normal hepatic architecture, impairs liver function, and may ultimately lead to cirrhosis or liver failure [237]. Persistent activation of hepatic stellate cells (HSCs) is a critical early event in this pathogenesis. Increased LIC directly correlates with HSC activation, evidenced by elevated *α*‐smooth muscle actin (*α*‐SMA) expression and enhanced collagen deposition in hereditary hemochromatosis patients [238, 239]. Physiologically, normal LIC is maintained below 32 μmol/g dry weight; however, exceeding 60 μmol/g disrupts cellular homeostasis, triggering HSC activation and subsequent ECM secretion [202, 240, 241]. Both in vivo and in vitro studies confirm that iron promotes pro‐fibrotic mediators in HSCs—including type I collagen and transforming growth factor‐*β* (TGF‐*β*)—thereby enhancing ECM synthesis [239]. Furthermore, iron accumulation in Kupffer cells stimulates pro‐inflammatory cytokine release. This indirectly activates HSCs and amplifies inflammatory responses to sustain fibrosis progression [242].

Cirrhosis, the terminal stage of chronic liver diseases (such as viral hepatitis and alcohol‐associated liver disease), is commonly accompanied by iron overload in hepatocytes and Kupffer cells. In animal models, particularly those using carbon tetrachloride (CCl_4_) to induce cirrhosis, exogenous iron supplementation markedly accelerates disease progression [243, 244]. Clinically, cirrhotic patients frequently exhibit elevated serum ferritin and hepatic iron deposition [245]. Concurrently, increased serum transferrin saturation (TS) serves as a robust positive predictor of cirrhosis and hepatocellular carcinoma (HCC) [246]. For example, in HCV‐induced cirrhosis, patients who progress to HCC exhibit significantly higher hepatic iron levels than those who remain HCC‐free [247].

### 4.6 Iron and Hepatocellular Carcinoma

Genetic mutations affecting iron metabolism and dietary iron overload are strongly linked to increased HCC incidence. As early as the mid‐19th century, dietary iron overload was reported in African populations consuming home‐brewed beer fermented in iron vessels, which produced excessively high iron concentrations [248]. As urbanization and socioeconomic development shifted consumption toward commercial beer, regional HCC incidence notably declined [249]. In Northern Europe, hereditary hemochromatosis (HH) drives abnormal hepatic iron accumulation; recent large‐scale cohorts indicate that approximately 1.5%–6% of these patients ultimately develop HCC [250, 251]. Similarly, dietary heme iron (abundant in red meat) positively correlates with HCC risk, yielding a relative risk of 1.22 (95% CI: 1.01–1.46) [252, 253]. Mechanistically, elevated intracellular iron promotes ROS generation, intensifies oxidative stress, and induces DNA damage. These factors disrupt cellular integrity and collectively facilitate the malignant transformation of hepatocytes [254, 255, 256, 257, 258]. Furthermore, iron overload dysregulates the cell cycle by upregulating *CCND1* (driving G1‐to‐S phase progression), thereby directly contributing to hepatocarcinogenesis [178].

The tumour suppressor p53, activated by DNA damage or oncogenic stress, is critically modulated by iron levels [259, 260]. Excess iron downregulates *p53* via heme‐mediated interactions with its DNA‐binding domain [261]. Conversely, iron chelation enhances *p53* activity by inducing target genes such as *p21* [262, 263]. This dynamic indicates that iron overload actively promotes carcinogenesis through *p53* suppression.

Animal studies provide profound mechanistic insights. Muto and colleagues generated the first genetically engineered mouse model of iron overload‐induced hepatocarcinogenesis: the *Fbxl5*‐deficient mouse. *Fbxl5* deletion causes IRP2 accumulation and hepatic iron overload. This cascade triggers oxidative stress, tissue injury, inflammation, and compensatory hepatocyte proliferation, ultimately enhancing diethylnitrosamine (DEN)‐induced HCC. Notably, concurrent *Irp2* deletion ameliorates tumourigenesis in these *Fbxl5*‐deficient mice, highlighting IRP2's pivotal role in iron‐mediated HCC development [185].

Clinically, excessive hepatic iron deposition in HH patients predicts poor prognosis across various liver diseases, accelerating the transition from hepatitis to fibrosis, cirrhosis, and ultimately HCC [212]. Although substantial progress has elucidated iron's broad role in liver pathology, the precise molecular mechanisms driving this disease progression remain incompletely understood.

## 5 Potential Mechanisms of Iron Metabolism Disorders

Intracellular iron accumulation frequently induces lipid peroxidation to trigger ferroptosis, a regulated cell death pathway increasingly implicated in liver disease progression. Furthermore, mitochondrial iron overload can precipitate localized ferroptosis and profound metabolic dysfunction. Collectively, these disruptions in iron homeostasis promote the initiation and exacerbation of liver pathologies through multiple interconnected molecular networks.

### 5.1 Ferroptosis in Liver Diseases

Ferroptosis is closely linked to MASLD development and progression. MASLD patients exhibit elevated circulating ferritin and lipid ROS. In dietary models of MASH, treatment with iron chelators (e.g., deferoxamine) reduces hepatocellular death and inflammatory cytokine infiltration [264, 265]. Furthermore, hepatic GPX4 levels are markedly increased in mice with MASH or type 2 diabetes (T2D), and macrophage ferroptosis is thought to facilitate the critical transition from MASLD to MASH [266, 267].

In mouse models of ALD, short‐term ethanol feeding rapidly induces hepatic injury. Crucially, treatment with the ferroptosis inhibitor Ferrostatin‐1 (Fer‐1) alleviates this liver toxicity [268]. Similarly, dimethyl fumarate protects against alcohol‐induced oxidative stress by activating *Nrf2* to suppress ferroptosis [269]. Moreover, intestinal *Sirt1* deficiency mitigates alcohol‐induced iron dyshomeostasis, reducing hepatic ferroptosis and protecting against liver injury [270]. Wild‐type mice inherently resist advanced alcoholic injury due to their rapid ethanol clearance. Consequently, short‐term feeding models only induce mild steatosis, failing to mimic the severe hepatitis and chronic fibrosis seen in humans [271, 272, 273].

Ferroptosis also critically impacts viral hepatitis. During HCV infection, ferroptosis restricts viral replication via FADS2‐dependent lipid peroxidation; conversely, iron chelation with deferoxamine eliminates this lipid peroxidation and paradoxically enhances HCV replication [274]. Supporting this antiviral role, in vitro studies using Huh7.5.1 hepatoma cells reveal that iron inhibits viral RNA and protein expression by suppressing NS5B RNA polymerase activity, thereby severely impairing HCV replication [275].

### 5.2 Mitochondrial Metabolic Dysregulation

Cellular iron accumulation markedly impairs mitochondrial Fe–S cluster and heme biosynthesis. Concurrently, it promotes ROS overproduction, which disrupts oxidative phosphorylation, induces lipid peroxidation, and alters mitochondrial fission dynamics. Collectively, these profound mitochondrial disturbances constitute core pathogenic mechanisms driving MASLD, ALD, and viral hepatitis.

#### 5.2.1 Mitochondrial Dysfunction in Liver Disease

Studies in mammals indicate that early mitochondrial dysfunction contributes to chronic inflammatory states in the liver. For instance, treating hepatocytes with NaAsO_2_ induces marked mitochondrial injury. This effect is linked to the inhibition of *Cisd2*, which drives intracellular iron redistribution and mitochondrial dysfunction, ultimately promoting MASH [276]. Genetic evidence further highlights the necessity of mitochondrial iron homeostasis in hepatic physiology. Concurrent deletion of *Irp1* and *Irp2* in hepatocytes causes mitochondrial iron deficiency and profound dysfunction. This disrupts ISC and heme biosynthetic pathways, resulting in liver failure and death [277]. Similarly, liver‐specific deletion of *Pcbp1* impairs mitochondrial structure and function. It elevates plasma ALT, reduces respiratory chain complex II and IV levels, and decreases oxygen consumption and ATP production, thereby driving hepatic steatosis, triglyceride accumulation, and liver injury [278].

Mitochondrial iron import, mediated by SLC25A37 and SLC25A28, is equally essential for hepatic homeostasis. SLC25A37 is required for erythroid development; its deficiency causes embryonic lethality and severe anaemia in adult mice [279]. Although liver‐specific loss of *Slc25a37* alone has a limited impact on overall development, its deletion in an *Slc25a28* ^−/−^ background markedly impairs liver regeneration [280]. Furthermore, whole‐body deletion of *Slc25a28* under dietary iron restriction exacerbates MASLD and disrupts lipid metabolism [281].

Both in vivo and in vitro studies demonstrate that iron overload directly induces mitochondrial injury and hepatocyte death. Intraperitoneal iron administration in mice increases ROS formation and mitochondrial swelling, prompting hepatocyte apoptosis [282]. Corroborating this, analysis of liver mitochondria from Fischer 344 × Brown Norway rats across different ages indicates that age‐dependent mitochondrial iron accumulation exacerbates dysfunction and oxidative damage, heightening susceptibility to apoptosis [283].

Mechanistically, iron overload severely compromises mitochondrial DNA (mtDNA) integrity. Cells exposed to high iron for 3–5 days exhibit cumulative mtDNA oxidative damage, reduced respiratory activity, and decreased mRNA levels of 16S rRNA alongside several mtDNA‐encoded electron transport chain subunits. Collectively, these factors impair respiratory chain assembly and mitochondrial function [284]. Moreover, mtDNA integrity is paramount for liver regeneration. In *Top1mt*‐deficient mice, mtDNA copy numbers fail to increase during recovery from partial hepatectomy or CCl_4_‐induced injury, resulting in reduced regeneration and impaired hepatocyte proliferation [285]. Accumulating mtDNA mutations also accelerate hepatocyte ballooning and promote premature organ aging [286]. Clinical observations robustly support this link between mitochondrial iron, mtDNA instability, and liver disease. Compared to healthy controls, patients with MASLD exhibit reduced hepatic mtDNA content, elevated mtDNA mutation rates (1.28‐fold), and increased heteroplasmy [287].

#### 5.2.2 Mitochondrial Dysfunction in Erythropoietic Protoporphyria

Mitochondria carry out the terminal steps of heme biosynthesis, and disruption of this pathway represents a critical mechanism of hepatic injury. Erythropoietic protoporphyria (EPP) exemplifies this process: mutations in the *FECH* gene reduce ferrochelatase activity, impairing the final step of heme synthesis and causing hepatic accumulation of protoporphyrin IX. Because this poorly soluble intermediate is inefficiently excreted via bile, it drives cholestasis and bile cast formation, thereby triggering severe hepatocellular damage and inflammatory responses [288]. Clinically, managing these severe cases often requires combined bone marrow and liver transplantation [289].

In summary, intracellular iron overload induces multidimensional cellular damage. It not only severely disrupts mitochondrial homeostasis—impairing oxidative phosphorylation alongside Fe–S cluster and heme biosynthesis—but also renders hepatocytes highly susceptible to ferroptosis. Together, these parallel and interacting pathological events (mitochondrial dysfunction and ferroptosis) exacerbate lipid peroxidation and serve as core mechanisms driving the progression of various liver diseases. Consequently, maintaining rigorous systemic iron homeostasis is of paramount physiological importance to halt liver pathogenesis.

## 6 Treatment of Iron‐Related Disorders

Given the central role of iron dysmetabolism—particularly iron overload and aberrant ferroptosis sensitivity—in driving hepatic pathogenesis, targeting iron homeostasis has emerged as a highly promising avenue for clinical intervention. Current therapeutic strategies generally encompass two parallel and complementary dimensions. The first focuses on systemic iron overload disorders, such as hereditary hemochromatosis (HH). These interventions aim to directly restore systemic and organ‐level iron balance through physical depletion, iron chelators, or the modulation of hepcidin signaling pathways. The second dimension targets a broad spectrum of stress‐induced acquired liver diseases. This approach utilizes pharmacological agents, small‐molecule compounds, and natural products to precisely modulate ferroptosis pathways. Consequently, these therapies can either protect vulnerable hepatocytes or selectively eliminate pathogenic cells, such as tumour cells and activated hepatic stellate cells. This chapter systematically reviews the latest clinical applications and frontier research advances of these dual therapeutic strategies across specific liver diseases.

### 6.1 Treatment of Hereditary Hemochromatosis Patients

Traditional treatments for HH include phlebotomy, erythrocytapheresis, and iron chelation therapy. Phlebotomy remains the most widely used and cost‐effective approach. Its induction and maintenance regimens are guided by serum ferritin levels. In general, baseline phlebotomy is initiated when ferritin exceeds 300 μg/L in men or 200 μg/L in women. Subsequently, most clinical guidelines recommend approximately 3–4 sessions per year to maintain target ferritin levels [290]. Erythrocytapheresis selectively removes red blood cells rather than whole blood. Although this technique requires specialized instrumentation, each procedure removes more iron and thereby reduces treatment frequency. Iron chelators may also be used adjunctively to facilitate systemic iron removal [291].

Beyond these established approaches, emerging small‐molecule agents have expanded therapeutic possibilities for HH. As patients with HH exhibit markedly reduced hepcidin levels, hepcidin replacement or stimulation remains a central therapeutic strategy. Nevertheless, the complex secondary structure and rapid turnover of native hepcidin limit its direct clinical application. This limitation has prompted the development of hepcidin‐mimetic peptides and agonists. The N‐terminal 7–9 amino acids of hepcidin are essential for SLC40A1 binding. These specific residues have been incorporated into stable, bioactive truncated derivatives termed minihepcidins. Ultimately, these minihepcidins effectively inhibit SLC40A1. In HH models, minihepcidins prevent hepatic iron deposition, promote duodenal iron retention, and redistribute iron from the liver to the spleen [292, 293, 294]. Clinically, this approach is exemplified by rusfertide (PTG‐300), a first‐in‐class hepcidin mimetic. Recent Phase 2 trials revealed that rusfertide effectively prevents iron re‐accumulation and stabilizes both transferrin saturation and serum iron levels in HH patients, thereby virtually eliminating the requirement for therapeutic phlebotomy [295].

TMPRSS6, a negative regulator of hepcidin, suppresses *HAMP* transcription. In murine models, suppressing *Tmprss6* via lipid nanoparticle‐delivered siRNA or second‐generation antisense oligonucleotides (ASOs) effectively increases hepcidin expression and reduces systemic iron accumulation [296, 297]. Advancing these RNA‐targeting strategies into clinical translation, two emerging GalNAc‐conjugated agents—the siRNA divesiran and the ASO sapablursen—are currently demonstrating promising preliminary efficacy by directly silencing hepatic *TMPRSS6*. By alleviating TMPRSS6‐mediated suppression, both agents effectively elevate endogenous hepcidin production. In recent early‐phase clinical trials involving patients with polycythemia vera, subcutaneous administration of these agents yielded sustained hepcidin surges, excellent haematocrit control, and a substantial reduction in the need for therapeutic phlebotomies [298, 299].

Upstream hepcidin regulators such as bone morphogenetic proteins (BMPs) also represent key therapeutic targets. For instance, recombinant BMP6 restores impaired BMP6‐SMAD signalling and enhances hepcidin production in hemochromatosis models [300]. Small‐molecule screening has identified additional hepcidin‐activating compounds. In zebrafish, progesterone and two steroid molecules (epitiostanol and mifepristone) increase hepcidin expression through BMP‐ and STAT3‐independent mechanisms [301, 302]. In contrast, genistein enhances STAT3 phosphorylation and induces *HAMP* expression in hepatocellular carcinoma cells [303]. Although these small‐molecule hepcidin agonists show promise, their specificity, safety, and clinical efficacy require further investigation before they can be applied to correct pathological hepcidin deficiency.

### 6.2 Targeting Ferroptosis for the Treatment of MASLD

To date, no definitive therapeutic strategy for MASLD has been established. Given the central involvement of lipid peroxidation and iron overload in disease progression, ferroptosis‐targeted interventions have emerged as promising approaches for MASLD, MASH, and related hepatic disorders. Clinically approved iron chelators—including deferoxamine (DFO), deferiprone (DFP), and deferasirox (DFX)—have been evaluated in experimental models of fatty liver disease. Intraperitoneal DFO administration reduces hepatic iron content and modulates lipid‐metabolism‐related proteins. Furthermore, this treatment mitigates hepatocellular injury and attenuates steatosis in *ob/ob* mice [304]. In MCD diet‐induced MASLD models, treatment with DFO or the ferroptosis inhibitors Lip‐1 and Fer‐1 alleviates MASH severity, hepatic inflammation, and the accumulation of lipid ROS and lipid droplets. In contrast, the ferroptosis inducer RSL‐3 produces opposite effects [267, 305]. Consistently, the ferroptosis inducers RSL‐3 and erastin exacerbate elevations in serum ALT/AST, hepatic steatosis, inflammation, and apoptosis in MASLD mice [267, 305, 306, 307].

Approved pharmacological agents have also been explored for their ferroptosis‐modulatory potential. Vitamin E (VE), an antioxidant, exerts therapeutic effects in MASLD [308]. Rosiglitazone inhibits ferroptosis by suppressing *ACSL4* expression through a PPAR‐γ‐independent mechanism [309]. Additionally, *Acsl4* knockdown reduces 5‐HETE levels. This reduction attenuates arsenic‐induced MASH and ferroptosis [307]. Several novel small molecules have shown efficacy as well. Zileuton, a selective 5‐LOX inhibitor, blocks ferroptosis by suppressing 5‐LOX activity. Consequently, it ameliorates MASLD by modulating the AA‐5‐LOX pathway [310, 311]. Ginkgolide B protects against MASLD by inhibiting ferroptosis via NRF2 pathway activation [312]. Dehydroabietic acid exerts anti‐ferroptotic effects by binding KEAP1 and activating the NRF2‐ARE axis. This activation improves MASLD phenotypes [313].

Beyond small molecules, non‐coding RNAs—including miRNAs and lncRNAs—play important roles in regulating iron homeostasis and iron metabolism dysregulation in MASLD [314]. miR‐200a and miR‐223 are upregulated in MASLD mice and may contribute to disturbances in iron handling [315]. Fructose‐induced ferroptosis has been reported to trigger the release of liver‐specific miR‐33 into the serum [316]. Additionally, the lncRNA MAYA has been proposed as a therapeutic target capable of ameliorating iron overload, mitochondrial damage, and hepatocyte senescence in MASLD [317].

### 6.3 Targeting Ferroptosis for the Treatment of ALD

Restoring iron homeostasis in ALD has emerged as a key strategy to slow disease progression. Evidence indicates that DFP effectively lowers hepatic iron overload, lipid peroxidation, and lipid accumulation in rats chronically exposed to ethanol [318]. Meanwhile, the novel iron chelator M30 markedly attenuates ethanol‐induced apoptosis, oxidative stress, and inflammatory responses [319]. Given that oxidative stress and ferroptosis constitute major downstream mechanisms of ethanol‐induced liver injury, antioxidants and ferroptosis inhibitors may offer substantial therapeutic benefit for ALD. Several antioxidants mitigate alcohol‐induced liver damage in animal models. Furthermore, Ferrostatin‐1 significantly reduces ethanol‐induced injury both in vitro and in vivo [268].

In addition to pharmacological inhibitors, multiple natural compounds exhibit hepatoprotective effects by stabilizing iron homeostasis. The flavonoid quercetin, which acts as an iron‐chelating antioxidant, alleviates ethanol‐induced liver injury [320, 321]. Similarly, dimethyl fumarate improves ALD outcomes in mouse models by suppressing lipid peroxidation and inhibiting hepatocyte ferroptosis [269].

### 6.4 Targeting Ferroptosis for the Treatment of Liver Fibrosis

Liver fibrosis is another condition in which ferroptosis‐based interventions show therapeutic promise. Activation of HSCs is a central driver of fibrogenesis. Consequently, inducing ferroptosis in activated HSCs exerts potent anti‐fibrotic effects. Inhibition of the cystine/glutamate antiporter SLC7A11 promotes HSC ferroptosis [322]. Furthermore, ferroptosis inducers such as erastin and sorafenib attenuate liver fibrosis in mice through this pathway [323]. Several natural compounds act similarly. For instance, wogonoside induces HSC ferroptosis via the SOCS1/P53/SLC7A11 axis [324]. In contrast, chrysophanol suppresses the SLC7A11 pathway to produce comparable effects [325]. Wild bitter melon extract triggers ferroptosis in activated HSCs by downregulating *GPX4* and *SLC7A11* [326]. Artesunate derivatives also alleviate fibrosis by inducing ferritinophagy‐mediated ferroptosis in HSCs [327]. Additional natural products—including curcumol and dihydroartemisinin (DHA)—promote HSC ferroptosis [328]. Similarly, celastrol exerts anti‐fibrotic activity through a comparable mechanism [329].

### 6.5 Targeting Ferroptosis for the Treatment of Viral Hepatitis

FADS2 has also been identified as a rate‐limiting determinant of ferroptosis. Emerging evidence suggests that pharmacological modulation of the ferroptotic pathway may suppress viral replication [274]. Several natural compounds—including luteolin‐7‐O‐glucoside, ethanolic extracts of mangosteen pericarp, and pycnogenol—have been reported to attenuate HBV‐ or HCV‐induced liver injury by targeting ferroptosis. Consequently, these compounds inhibit viral replication and reduce ROS accumulation [330, 331, 332].

### 6.6 Targeting Ferroptosis for the Treatment of Liver Cancer

Ferroptosis and its inducers have been widely investigated in HCC and are increasingly recognized as an important tumour‐suppressive mechanism [333]. Conversely, repression of ferroptosis‐related genes enhances chemoresistance in HCC cells [334]. Sorafenib, a first‐line targeted therapy for advanced HCC, induces the expression of metallothionein‐1G (*MT‐1G*). The upregulation of *MT‐1G* acts as a negative regulator of ferroptosis. Consequently, this upregulation contributes to sorafenib resistance [335]. In contrast, the ferroptosis‐promoting enzyme ACSL4 enhances iron‐dependent cell death. Clinical evidence indicates that elevated ACSL4 protein abundance in resected HCC specimens correlates with improved postoperative responses to sorafenib [336, 337]. Lenvatinib, another multi‐kinase inhibitor used in advanced HCC, exerts antitumour activity by inducing ferroptosis through inhibition of the system X_c_^−^ pathway [338]. Ceruloplasmin has been reported to suppress ferroptosis in HCC cells. Conversely, its downregulation promotes intracellular iron accumulation and ROS generation. This process sensitizes cells to erastin‐induced ferroptosis [339]. The lactate‐rich microenvironment characteristic of HCC enhances monounsaturated fatty acid synthesis and suppresses ferroptosis via monocarboxylate transporter 1 (*MCT1*)‐dependent lactate uptake. Accordingly, inhibition of *MCT1* increases ferroptosis sensitivity. Natural polyunsaturated fatty acids (PUFAs), particularly *ω*‐3 PUFAs, constitute major lipid‐peroxidation substrates for ferroptosis. They display notable antitumour potential, and increased dietary intake is associated with reduced HCC risk [340, 341]. In addition, heteronemin—a marine‐derived terpenoid—induces ferroptosis in HCC cells by downregulating GPX4 [342]. Furthermore, interferon‐γ (IFN‐γ) promotes ferroptosis by suppressing system X_C_^−^ activity [343].

### 6.7 Targeting Mitochondria for the Treatment of Liver Diseases

The onset of insulin resistance (IR) is tightly linked to mitochondrial dysfunction and represents an early feature of MASLD. Perfluorooctane sulfonate (PFOS) has been shown to induce abnormal mitochondrial iron accumulation in mouse liver and human hepatocytes (L‐O2). In PFOS‐treated L‐O2 cells, mitochondrial iron overload precedes the development of IR. Removal of excess mitochondrial iron using iron chelators effectively ameliorates PFOS‐induced IR [344]. This finding suggests that perturbations in mitochondrial iron homeostasis may serve as a key mechanistic link between mitochondrial dysfunction and IR.

Given the central role of mitochondrial iron regulation in metabolic dysregulation and cell death, molecular engineering strategies aimed at precisely modulating intracellular iron flux have been explored. Ferritin is highly stable under physiological conditions and releases minimal iron. Exploiting this inherent stability, researchers have developed a self‐triggered proteolytic RNA interference system (cRGD‐VFs). Once deployed, the engineered nanoplatform selectively targets mitochondria to restore iron homeostasis and activate antitumour immunity. By suppressing ENO1 transcription, it modulates the ENO1‐IRP1‐Mfrn1 axis, upregulates *Mfrn1*, and enhances mitochondrial iron import. The resulting iron accumulation drives ROS generation, triggers oxidative stress and mitochondrial dysfunction, disrupts redox homeostasis, and ultimately induces tumour cell death [345].

Recent studies further demonstrate that mitochondrial iron overload and ROS production are closely associated with ferroptosis and hold important implications for HCC pharmacotherapy. During sorafenib‐induced ferroptosis, loss of the mitochondrial acetylation regulator GCN5L1 decreases transcription of *CISD1* and *SLC40A1*, impairs iron efflux, and promotes mitochondrial iron accumulation. This cascade amplifies ROS production and enhances sorafenib sensitivity. Conversely, *CISD1* overexpression facilitates mitochondrial iron export and maintains mitochondrial integrity. Notably, acute sorafenib treatment suppresses *GCN5L1* expression. In contrast, sorafenib‐resistant HCC cells display elevated GCN5L1 levels. Co‐administration of the CISD1 inhibitor NL‐1 with sorafenib markedly enhances antitumour efficacy. This indicates that targeting mitochondrial iron metabolism offers a promising strategy to overcome drug resistance and improve therapeutic outcomes in HCC [346].

## 7 Conclusion

Iron is an essential trace element critical for cellular respiration and serves as a cofactor for key enzymes, such as DNA polymerase and catalase. Furthermore, iron regulates macrophage polarization and the differentiation of various immune cell lineages [51, 57, 60, 347, 348, 349, 350]. Maintaining iron homeostasis is therefore indispensable for normal physiological function. The liver acts as the primary iron storage organ and the central regulator of systemic iron balance.

Disruption of this delicate iron homeostasis contributes to the onset and progression of a wide range of liver diseases, including MASLD, MASH, ALD, liver fibrosis, and viral hepatitis. Chronic iron overload increases the risk of hepatocellular carcinoma, particularly in individuals with HH. Hepatic injury in these conditions is closely linked to abnormal iron accumulation in the cytosol and mitochondria. Excess iron drives ROS production, impairs mitochondrial function, promotes lipid peroxidation, and triggers ferroptosis. Ultimately, these cascades accelerate liver pathology [241, 246, 351, 352, 353, 354].

Unresolved diagnostic gaps severely hinder the clinical management of hepatic iron overload. Standard screening relies on serum ferritin, which severely lacks specificity due to ubiquitous confounding by systemic inflammation and metabolic dysfunction (MASLD) [355]. Advanced imaging modalities like MRI R_2_* relaxometry provide non‐invasive iron quantification, though their diagnostic precision and widespread implementation are frequently compromised by concurrent severe hepatic steatosis, high costs, and limited specialized software [202, 206, 207]. Overcoming these clinical limitations necessitates the rigorous validation of more precise, dynamic biomarkers like soluble transferrin receptor (sTfR) and labile plasma iron (LPI). Cleaved from the membrane protein TfR1, circulating sTfR serves as a highly specific serum biomarker reflecting real‐time cellular iron demand. A complete lack of assay standardization precludes the establishment of universal sTfR reference ranges [356]. As a highly redox‐active sub‐component of non‐transferrin‐bound iron (NTBI), LPI permeates cells via free ion channels to dangerously elevate toxic LIP. The clinical translation of these mechanistic insights is currently stalled. Assays for NTBI and LPI lack standardization and remain strictly confined to specialized research facilities [357]. Furthermore, evaluating specific serum hepcidin isoforms presents a unique diagnostic frontier. Standard immunoassays only quantify total hepcidin, failing to distinguish the bioactive hepcidin‐25 from its inactive truncated degradation products (e.g., hepcidin‐20, ‐22, ‐24). Resolving these specific isoforms via mass spectrometry offers a highly targeted, albeit clinically unvalidated, approach to refine iron toxicity assessments and guide therapeutic decisions [358, 359].

Current therapeutic strategies exhibit profound clinical limitations. Traditional treatments, including phlebotomy and non‐specific iron chelators (e.g., deferoxamine, deferasirox), carry significant adverse effects and organ toxicity profiles, restricting their routine application [291, 360, 361]. Consequently, targeted pharmacological interventions are under intense investigation, yet they face significant translational hurdles. Hepcidin mimetics aim to restore the primary iron‐regulatory axis; their clinical translation is severely hindered by short half‐lives, a dangerously narrow therapeutic window risking iron‐restricted erythropoiesis, and off‐target pleiotropic effects [362].

Similarly, while ferroptosis modulators (such as Ferrostatin‐1) demonstrate profound efficacy in preclinical rodent models [268, 363], their application in humans faces severe experimental constraints. Critical challenges include poor in vivo pharmacokinetic profiles, a lack of liver‐targeted delivery systems, and the inherent risk of broadly suppressing physiological ferroptosis required for tumour immune surveillance [364].

Advancing translational research requires overcoming the inherent limitations of current experimental models. Standard transgenic rodents, such as *Hfe*‐knockout mice, fail to fully recapitulate the chronic, multifactorial pathogenesis and complex immune‐iron crosstalk of human liver disease. Furthermore, precise mechanistic insights are constrained by a scarcity of high‐resolution spatial mapping tools for labile ferrous iron, the most reactive and unstable intracellular iron species. Future translational efforts must prioritize the development of humanized liver chimeras and patient‐derived liver organoids to better mirror human pathology. Integrating these advanced models with highly specific, subcellular iron‐sensing probes and liver‐targeted delivery systems will be essential to translate molecular discoveries into precision diagnostics and therapeutics for iron‐related liver diseases.

## Author Contributions

**Peng Yang:** writing – original draft, writing – review and editing, conceptualization. **Zhitian Shi:** writing – original draft, writing – review and editing. **Qin Huang:** writing – original draft, writing – review and editing. **Zhihong Tu:** writing – original draft, writing – review and editing. **Yue Sun:** writing – review and editing, supervision, funding acquisition, conceptualization, validation. **Lin Wang:** writing – review and editing, supervision, funding acquisition, conceptualization, validation. **Yonglong Wei:** writing – review and editing, supervision, funding acquisition, conceptualization, validation.

## Conflicts of Interest

The authors declare no conflicts of interest.

## Data Availability

No new datasets were generated or analyzed in this review. All information discussed is derived from published literature cited within the reference list.
